# Rewired functional regulatory networks among miRNA isoforms (isomiRs) from let-7 and miR-10 gene families in cancer

**DOI:** 10.1016/j.csbj.2020.05.001

**Published:** 2020-05-13

**Authors:** Tingming Liang, Leng Han, Li Guo

**Affiliations:** a Jiangsu Key Laboratory for Molecular and Medical Biotechnology, School of Life Science, Nanjing Normal University, Nanjing 210023, China; b Department of Biochemistry and Molecular Biology, The University of Texas Health Science Center at Houston McGovern Medical School, Houston, TX 77030, USA; c Department of Bioinformatics, Smart Health Big Data Analysis and Location Services Engineering Lab of Jiangsu Province, School of Geographic and Biologic Information, Nanjing University of Posts and Telecommunications, Nanjing, China

**Keywords:** ACC, adrenocortical carcinoma, BLCA, bladder urothelial carcinoma, BRCA, breast invasive carcinoma, CESC, cervical squamous cell carcinoma and endocervical adenocarcinoma, CHOL, cholangiocarcinoma, COAD, colon adenocarcinoma, ESCA, esophageal carcinoma, GBM, glioblastoma multiforme, HNSC, head and neck squamous cell carcinoma, KICH, kidney chromophobe, KIRC, kidney renal clear cell carcinoma, KIRP, kidney renal papillary cell carcinoma, LAML, acute myeloid leukemia, LIHC, liver hepatocellular carcinoma, LGG, brain Lower grade glioma, LUAD, lung adenocarcinoma, LUSC, lung squamous cell carcinoma, MESO, mesothelioma, OV, ovarian serous cystadenocarcinoma, PAAD, pancreatic adenocarcinoma, PCPG, pheochromocytoma and paraganglioma, PRAD, prostate adenocarcinoma, READ, rectum adenocarcinoma, SARC, sarcoma, SKCM, skin cutaneous melanoma, STAD, stomach adenocarcinoma, TGCT, testicular germ cell tumors, THCA, thyroid carcinoma, THYM, thymoma, TSG, tumor suppressor gene, UCEC, uterine corpus endometrial carcinoma, UCS, uterine carcinosarcoma, UVM, uveal melanoma, MicroRNA (miRNA), IsomiR, Let-7, miR-10, Network, Function

## Abstract

Classical microRNA (miRNA) has been so far believed as a single sequence, but it indeed contains multiple miRNA isoforms (isomiR) with various sequences and expression patterns. It is not clear whether these diverse isomiRs have potential relationships and whether they contribute to miRNA:mRNA interactions. Here, we aimed to reveal the potential evolutionary and functional relationships of multiple isomiRs based on let-7 and miR-10 gene families that are prone to clustering together on chromosomes. Multiple isomiRs within gene families showed similar functions to their canonical miRNAs, indicating selection of the predominant sequence. IsomiRs containing novel seed regions showed increased/decreased biological function depending on whether they had more/less specific target mRNAs than their annotated seed. Few gene ontology(GO) terms and Kyoto Encyclopedia of Genes and Genomes (KEGG) pathways were shared among the target genes of the annotated seeds and the novel seeds. Various let-7 isomiRs with novel seed regions may cause opposing drug responses despite the fact that they are generated from the same miRNA locus and have highly similar sequences. IsomiRs, especially the dominant isomiRs with shifted seeds, may disturb the coding-non-coding RNA regulatory network. These findings provide insight into the multiple isomiRs and isomiR-mediated control of gene expression in the pathogenesis of cancer.

## Graphical abstract

**Figure:**
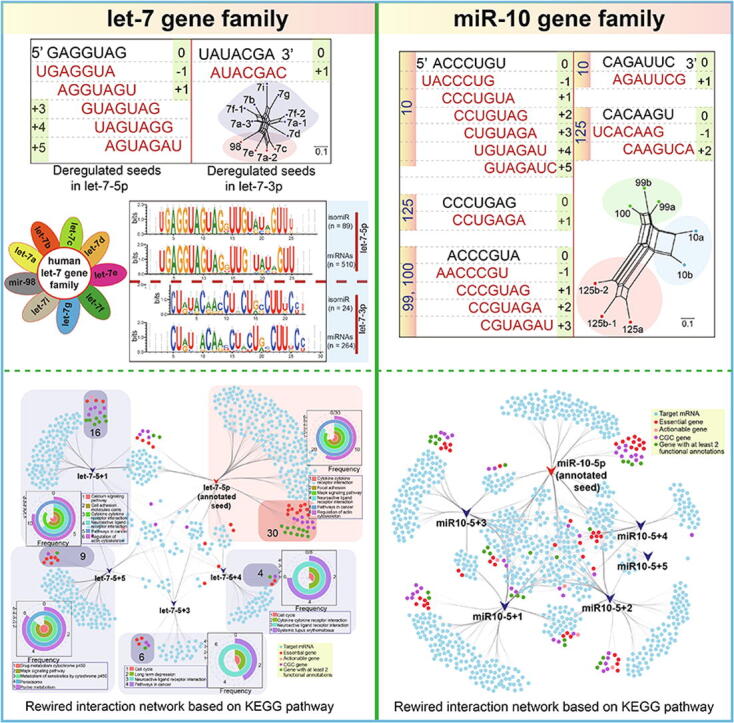

## 1 Introduction

MicroRNA (miRNA, about 22-nt) plays important regulatory roles in multiple biological processes by negatively regulating gene expression at the transcriptional or posttranscriptional levels [1], [2]. These small non-coding RNA molecules are also promising biomarkers for the diagnosis of cancer. Now, an increasing number of studies suggest that there are multiple varieties of miRNAs, termed isomiRs, which have divergent sequences and expression patterns. These diverse miRNA isoforms can be generated by nucleotidyl transferases, RNA editing during the miRNA maturation process [3], which simultaneously leads to sequence variation (especially at the 3ʹ end) and complex small RNA and mRNA interactions. The specificity of miRNAs is largely the result of binding of the miRNA seed region (nucleotides 2–8) to the 3ʹ UTR of the targeted mRNAs [4], [5]. The variation introduced by miRNA isoforms contributes to a dynamic microRNAome and rewiring of the coding-non-coding RNA regulatory network [6]. Therefore, the expression, function, and evolution of isomiRs is closely correlated with their clustered and/or homologous miRNA genes, implicating a potential synergistic effect of the diverse isomiRs in the regulatory network of a miRNA locus.

miRNAs from the same gene family or gene cluster have been studied and shown to have a similar function, such as those from the let-7 gene family [7], [8] and the miR-17-92 gene cluster [9], [10]. Several studies have focused on the multiple isomiRs that act as regulatory molecules [11], [12]. The most discriminatory isomiRs happen to also be differentially expressed between normal tissue and cancer, and they can successfully classify datasets from 32 cancers [13]. Furthermore, some isomiRs exhibit cancer heterogeneity [14] and gender-dependent expression profiles [15]. The abundance profiles of isomiRs and tRNA-derived fragments associate with various molecular phenotypes, metastatic disease, and patient survival in uveal melanoma [16]. It is still unclear whether regulatory networks are disrupted by the varied miRNA isoforms. Based on the potential expression and functional correlations among isomiRs that are similar to their homologous and/or clustered miRNAs, it is important to investigate potential relationships between isomiRs and homologous and/or clustered miRNAs. These relationships will be crucial in exploring miRNA diversity, the biological roles of isomiRs, and the importance of isomiR formation during the miRNA maturation process.

In this study, we selected the let-7 gene family to discuss potential relationships between isomiRs and their homologous and/or clustered miRNAs. Firstly, the let-7 gene family is an ancient family of miRNAs initially discovered as a heterochronic gene in *Caenorhabditis elegans* [17]. Let-7 miRNAs have been widely and comprehensively studied because of their important biological roles. For example, they can regulate developmental timing in *C. elegans*, control cardiomyocyte metabolism, and adjust cell size, among other functions [8], [17], [18], [19], [20], [21], [22], [23]. Secondly, let-7 is the largest among all human miRNA gene families, with nine homologous miRNA genes, and has a close relationship with the mir-10 gene family (some members of the two families cluster together, with an inter-miRNA distance of <10,000 bp), which we analyzed simultaneously in this study. Finally, based on the range of experimentally validated and predicted target mRNAs, let-7 miRNAs have a broader mRNA interaction network compared with other gene families, indicating that it is a crucial gene family. Therefore, we selected the let-7 gene family and its related miR-10 gene family to analyze expression and functional networks at the miRNA and isomiR levels, and to comprehensively discuss miRNA diversity based on the isomiRs and miRNAs involved in human cancers.

## 2 Materials and methods

### 2.1 Data source

The sequences of miRNAs and pre-miRNAs from the let-7 and miR-10 gene families were obtained from the miRBase database (version 22.1) [24]. The corresponding relationships among miRNAs, including physical relationships that mainly presented as miRNA gene clusters, were determined based on gene distributions (inter-miRNA distance < 10,000 bp).

To understand isomiR expression profiles in the two related gene families, we selected small RNA sequencing data from The Cancer Genome Atlas (TCGA, https://www.cancer.gov/tcga) to perform expression analysis using the R package TCGAbiolinks [25]. The sequence information of isomiR types across different cancers in the study are showed in Table S1. The target mRNAs of abnormally-expressed isomiRs were further queried for association with published cancer hallmark genes (http://software.broadinstitute.org/gsea/msigdb/) [26], Cancer Gene Census (CGC) (http://cancer.sanger.ac.uk/census) [27], core essential genes (essential genes from Hart et al. [28], Blomen et al. [29], and Wang et al. [30]), and tumor suppressor genes (TSG) and oncogenes [31]. The regulation of transcription factors and miRNAs was predicted using TransmiR version 2.0 [32].

### 2.2 Sequence and evolutionary analysis

Firstly, to understand the distribution of genes from the two gene families, particularly that of relevant gene clusters, the relative genomic locations of miRNA genes were estimated using chromPlot [33]. Secondly, relevant homologous miRNAs and their pre-miRNA sequences were aligned using multiple alignment program ClustalX 2.1 [34], while phylogenetic trees were generated based on the sequences of the miRNAs from the two gene families using the neighbor-joining (NJ) method in MEGA 7.0 [35], and the Neighbor-Net method in SplitsTree 4.14 [36]. Phylogenetic networks for the miRNAs and some isomiRs were constructed using Network software via the median-joining (MJ) method. Finally, to understand sequence features among multiple isomiRs and miRNAs from different animal species, sequence logos were constructed using WebLogo version 3.6.0 [37].

### 2.3 Expression analysis of the small RNAs and target mRNAs

To determine the expression profiles of abnormal isomiRs and mRNAs and examine potential interactions between them, we used DESeq2 [38] to obtain deregulated RNAs. Small RNA or mRNAs were classified as abnormal if the |log_2_FC| value was >1.5 and the adjusted p-value was <0.05. Further functional analyses were then performed for these abnormal isomiRs. Genes were eliminated from further analyses if they were detected in <50% of all samples. Repeated isomiRs from multicopy pre-miRNAs or homologous miRNAs were also eliminated from further sequence and function analyses.

### 2.4 Prediction and validation of target mRNAs

To understand the potential biological functions of isomiRs from the two gene families, abnormal isomiRs and annotated miRNAs (based on their novel seeds and annotated seeds, respectively) were used to predict their target mRNAs using TargetScan 7.0 [39]. The predicted targets were used in further analyses if the context score values were less than −0.40. The abnormally-expressed target mRNAs were then screened based on their deregulated mRNA expression profiles to identify isomiR:mRNA pairs where the two members showed opposing expression patterns in specific types of cancer (i.e., one was up-regulated while the other was down-regulated). Finally, the selected isomiR:mRNA pairs were further queried for expression correlations by co-expression analysis (*n* ≥ 50 for both the tumor and normal control groups), and candidate target mRNAs were identified based on a negative Spearman correlation coefficient and a false discovery rate (FDR) value of <0.05.

To further identify novel seeds with significantly different target mRNAs (based on candidate target mRNAs), we used a hypergeometric test to distinguish potential differences between the target mRNAs of novel and annotated seeds:

$$p\left(x,y\right)=F\left(N_{x,y}-1|N,N_{x},N_{y}\right)$$

$$=\sum_{t=0}^{\mathrm{Nx},y-1}\frac{\left(\begin{array}{c} N_{x}\\ t \end{array}\right)\left(\begin{array}{c} N-N_{x}\\ N_{y}-t \end{array}\right)}{\left(\begin{array}{c} N\\ N_{y} \end{array}\right)}$$

where *N_x,y_* was the number of mRNAs shared by novel and annotated seeds, *N* was the total number of involved target mRNAs, and *N_x_* and *N_y_* were the number of targets of the novel and canonical seeds, respectively. Differences were considered significant at p < 0.05, and identified novel seeds were further analyzed to examine potential functional differences compared with their annotated seeds.

### 2.5 Functional, survival, and drug response analyses

To further validate predicted functional differences between the target mRNAs of the novel and annotated seeds, candidate mRNAs were subjected to functional enrichment analysis using DAVID version 6.8 (The Database for Annotation, Visualization and Integrated Discovery) [40], and queried for association with cancer hallmark [26], clinically-actionable genes [41], CGC [27], and core essential genes [28], [29], [30]. Interaction networks of isomiR:mRNA pairs were presented using Cytoscape 3.6.0 [42].

Survival analysis was performed to identify potential differences between isomiRs with length and sequence divergence. A log-rank test was used to assess differences between two groups that were divided based on the median value of a specific isomiR type, with significance accepted at p < 0.05.

Finally, to further understand difference in drug response as a result of the diverse isomiRs, drug response analysis was performed using data available from the Genomics of Drug Sensitivity in Cancer (GDSC) database [43]. Drug sensitivity or resistance was accepted at |DF| > 0.1 and p < 0.05.

#### 2.6 Statistical analysis

Hypergeometric, wilcoxon rank-sum test and trend tests were used to estimate significant differences. All statistical analyses were performed using R version 3.4.3.

## 3 Results

### 3.1 Overall expression patterns of isomiRs from the let-7 and miR-10 families

Although let-7 and miR-10 miRNAs were always clustered together on chromosomes, they displayed divergent evolutionary patterns and differences in distribution were observed across different animal species. To further understand the expression patterns of the various homologous miRNAs between the two related gene families, we performed a comprehensive expression analysis using public human high-throughput sequencing data (TCGA data).

**Fig. 1:**
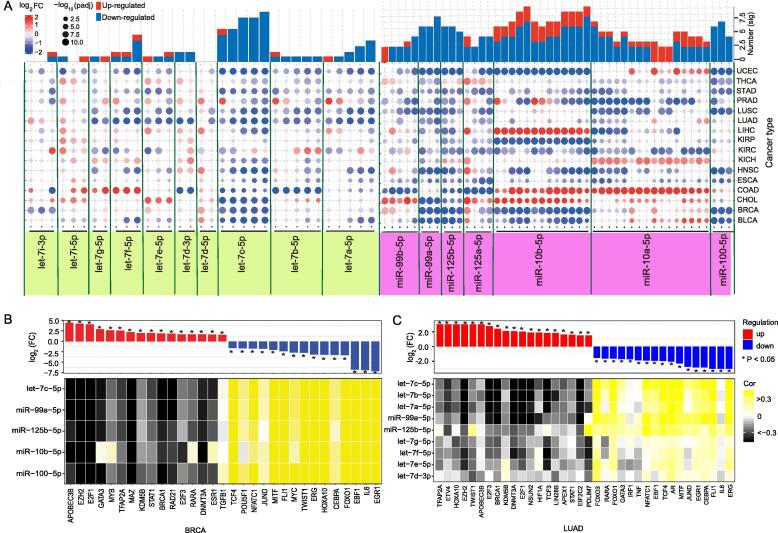
IsomiR repertoires of the let-7 gene family and related family miR-10 in 16 cancers. (A) Expression patterns of selected abnormal isomiRs from the two gene families across 16 cancer types (isomiRs with an RPM value not less than 50 and a baseMean value not less than 500 in at least one cancer type were selected). The log_2_FC distribution was estimated based on the original log_2_FC values. The numbers of deregulated isomiRs are also presented (number (sig.)). (B) Correlations among related transcription factors (TF) and miRNAs from the two gene families in BRCA patients (*n* = 1,207). (C) Correlations among related transcription factors (TF) and miRNA from the two gene families in LUAD patients (n =567).

Small RNAs were analyzed based on a detailed miRNA locus. Abnormally-expressed isomiRs from the two miRNA families in tumor samples were identified, and diverse expression patterns were detected for each miRNA locus and cancer type (Fig. 1A and Supplementary Fig. S1). IsomiR types and seed numbers varied across different loci and across cancer types (Supplementary Fig. S2A). No significant differences in the number of isomiRs were detected between the tumor samples and normal samples using a Wilcoxon rank-sum test (p = 0.4273 for the let-7 family and p = 0.4260 for the miR-10 family), indicating that isomiRs are stably transcribed and processed. The results also showed that isomiRs from the let-7 gene family were expressed relatively uniformly across the cancer types. In comparison, isomiRs from the miR-10 family, namely including miR-99b-5p, miR-125a-5p, miR-10b-5p, miR-10a-5p, showed inconsistent expression patterns across the cancer types (Fig. 1A and Supplementary Fig. S1).

IsomiR types and seed numbers also varied across different loci in breast invasive carcinoma (BRCA) patients (Supplementary Figs. S2B). miRNA let-7b-5p showed the largest number of isomiR types and seed types in the let-7 family, while for the miR-10 family, miR-10a-5p and miR-10b-5p showed an unusually large number of isomiR types and seed numbers compared with other miRNAs. The variations among loci were related to total enrichment levels at the specific locus, while variations among cancer types showed tissue-specificity and identified potential disease-associated small RNAs that may aid in screening and validating disease-associated isomiRs. Based on analysis of individual miRNA genes (pre-miRNA), we found that homologous miRNAs showed divergent expression levels in BRCA patients (*n* = 1,207) (p < 2.20e−16 for the let-7 family and p = 4.96e−05 for the miR-10 family, Supplementary Fig. S2C). Briefly, let-7b showed the highest level of expression (>10%), while miR-98 had the lowest level (<5%). Overall, miR-10-family miRNAs had higher average expression levels compared with let-7-family miRNAs (Supplementary Fig. S2C). Furthermore, the distribution of expression of homologous miRNAs of the let-7 and miR-10 families revealed that let-7a-3 and let-7b were the most highly expressed miRNAs from the let-7 family in BRCA. miR-10a, miR-10b, and miR-99 were the most highly expressed among the miR-10-family miRNAs (Supplementary Fig. S2D). These results indicated that miRNA enrichment levels are regulated by their precursor molecules.

Overall, reduced let-7 and miR-10 expression levels were frequently observed in 16 cancer types. We also identified a correlation between transcription factors (TF) and miRNAs from the two gene families in BRCA and LUAD patients. The results indicated that over-expression of a TF was always negatively correlated with the expression of its target miRNAs, while decreased expression of a TF was positively correlated with the expression of its target miRNAs (Fig. 1B and C). These findings may explain the reduced expression of let-7 and miR-10 family members in to some extent. In addition, opposing expression patterns in different cancer types (as was observed for let-7f-5p, miR-10a-5p, and miR-10b-5p) is indicative of cancer-specific expression of the small RNAs, while isomiRs with varied sequences but identical seed and functional regions would further complicate cross-talk between small RNAs and mRNAs.

### 3.2 Characterization of isomiR sequences and potential phylogenetic relationships

**Fig. 2:**
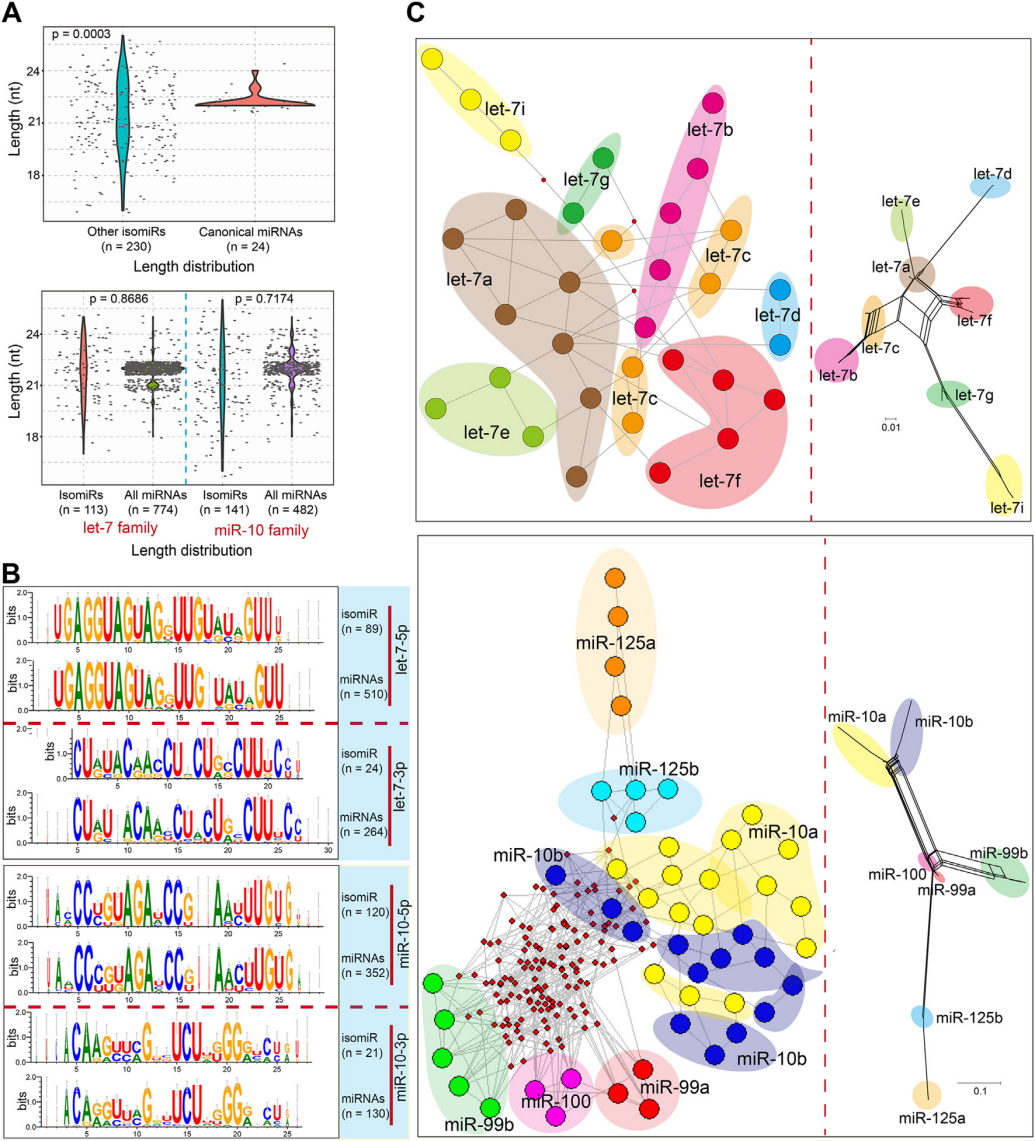
Correlations between internal isomiRs and external miRNAs, and phylogenetic relationships among isomiRs. (A) Length distributions of canonical miRNAs and their isomiRs from the two gene families, and length distributions of human isomiRs compared with mature miRNAs across animal species. (B) Sequence features of isomiRs and canonical miRNAs from the two gene families across animal species (5p and 3p were estimated). (C) Phylogenetic networks and trees of the most highly expressed isomiRs from 5p. IsomiRs with a RPM value ≥ 500 in any one sample were selected.

Typically, only annotated miRNAs are included in analyses, with other isomiRs tending to be ignored. Here, we analyzed the divergence between canonical miRNAs and other isomiRs to identify the potential roles of the ignored isomiRs. As expected, non-annotated isomiRs showed larger distribution intervals compared with their corresponding canonical miRNAs (p = 0.0003, Fig. 2A; p = 0.0088, Fig. S3A, based on isomiRs in Fig. 1A), with the isomiRs showing both sequence and length heterogeneities. Further analysis detected similar length distribution differences between all human isomiRs and all annotated canonical miRNAs (p = 0.8686 for the let-7 family and p = 0.7147 for the miR-10 family, Fig. 2A). Sequence analysis also showed similar sequence features between human isomiRs and annotated miRNAs, especially the highly-conserved miR-5p (Fig. 2B and Supplementary Fig. S3B). These similar distribution patterns were mainly caused by well-conserved features among the small RNAs, including among homologous miRNAs in the same species (such as homologous miRNAs in a gene family) and miRNAs across different animal species.

To determine the phylogenetic relationships among multiple human isomiRs, we attempted to construct a phylogenetic network using the MJ method. Compared with the network for isomiRs from the let-7 gene family, the network for isomiRs from the miR-10 family contained a greater number of hypothetical medians, and the corresponding network of annotated miRNAs also showed large genetic distances between miR-125, miR-99, and miR-100 and miR-10 (Fig. 2C and Supplementary Fig. S3C).

### 3.3 Varied seed regions regulate the diversity of target mRNAs

**Fig. 3:**
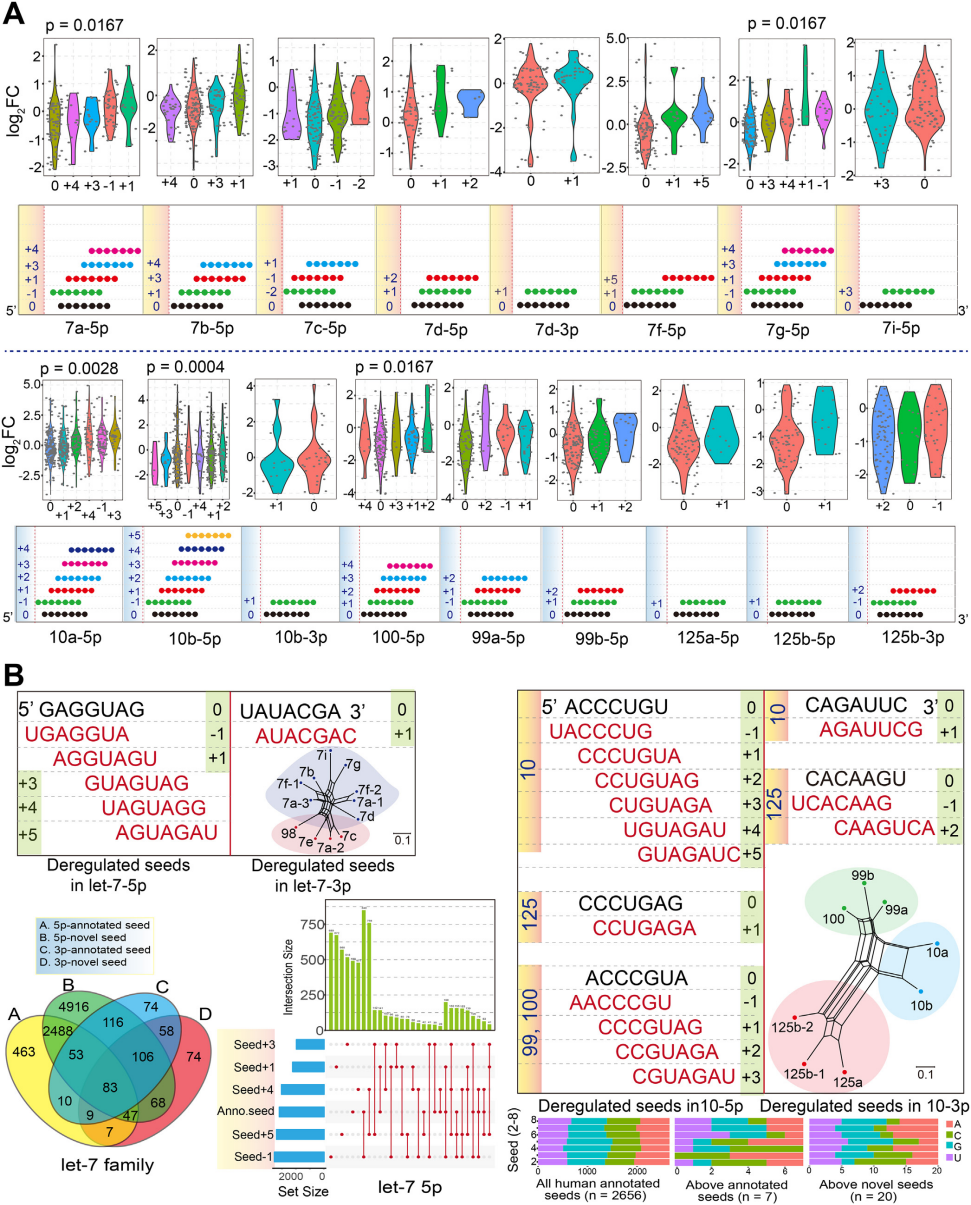
Seed variation among isomiRs. (A) Seed variations (annotated seed ± 5 nucleotides) and their deregulated expression patterns based on each miRNA locus. For multiple miRNAs, miRNAs are selected only if they had more isomiR types to avoid repeated estimation. Deregulated expression is estimated based on all relevant cancer types. If an isomiR shows decreased expression level, it will be removed from this analysis. miRNA loci are eliminated if no variations in the seed sequence are detected. (B) Detailed screening of deregulated seed sequences from the two miRNA gene families (a seed was is included if the corresponding isomiR is abnormally expressed in at least one cancer type), and distributions of predicted target mRNAs of annotated and novel seeds.

Our analysis showed that some isomiRs contained novel seed regions that arose via seed shifting events such as alternative and imprecise cleavage during the miRNA maturation process. These changes predominantly involved shifts in the 5ʹ or 3ʹ ends of the seed region, particularly the latter (Fig. 3A and Supplementary Fig. S4A). These novel isomiRs with shifted seeds showed various expression patterns, with some miRNA loci showing significant differences among shifted seeds based on log_2_FC values (p < 0.05 based on trend test, Fig. 3A). The two related gene families showed dominant expression at 5p loci, with fewer 3p loci showing abundant enrichment levels. Interestingly, we found that some novel seed sequences were consistent with other miRNAs. For example, the novel seed sequence of let-7-5p was identical to that of annotated miRNA miR-196-5p (Supplementary Fig. S4A).

To understand divergence in the targets of novel seeds versus annotated seeds, we further screened deregulated seeds to perform functional analyses. For the let-7 family, well-conserved homologous genes were only associated with annotated seeds in dominant 5p loci. In comparison, for the miR-10 family, only three annotated seeds in dominant 5p loci from three clusters were identified (Fig. 3B). These novel seeds had several specific target mRNAs that differed from those of their annotated seeds, despite also sharing some common targets. Similar distribution patterns could be detected among different seeds (distribution in let-7, Fig. 3B; distribution in miR-10, Supplementary Fig. S4B), indicating that novel seeds may target novel mRNAs compared with their annotated seeds. Moreover, similar nucleotide compositions in the seed region were identified in all annotated human seeds and all deregulated novel seeds, although these novel seeds were shifted at the 5ʹ or 3ʹ ends (Fig. 3B). This finding indicates that novel seeds have a similar nucleotide distribution, which further ensures their potential interactions with target mRNAs.

### 3.4 Regulatory network rewiring showing function correlations in signaling pathways

**Fig. 4:**
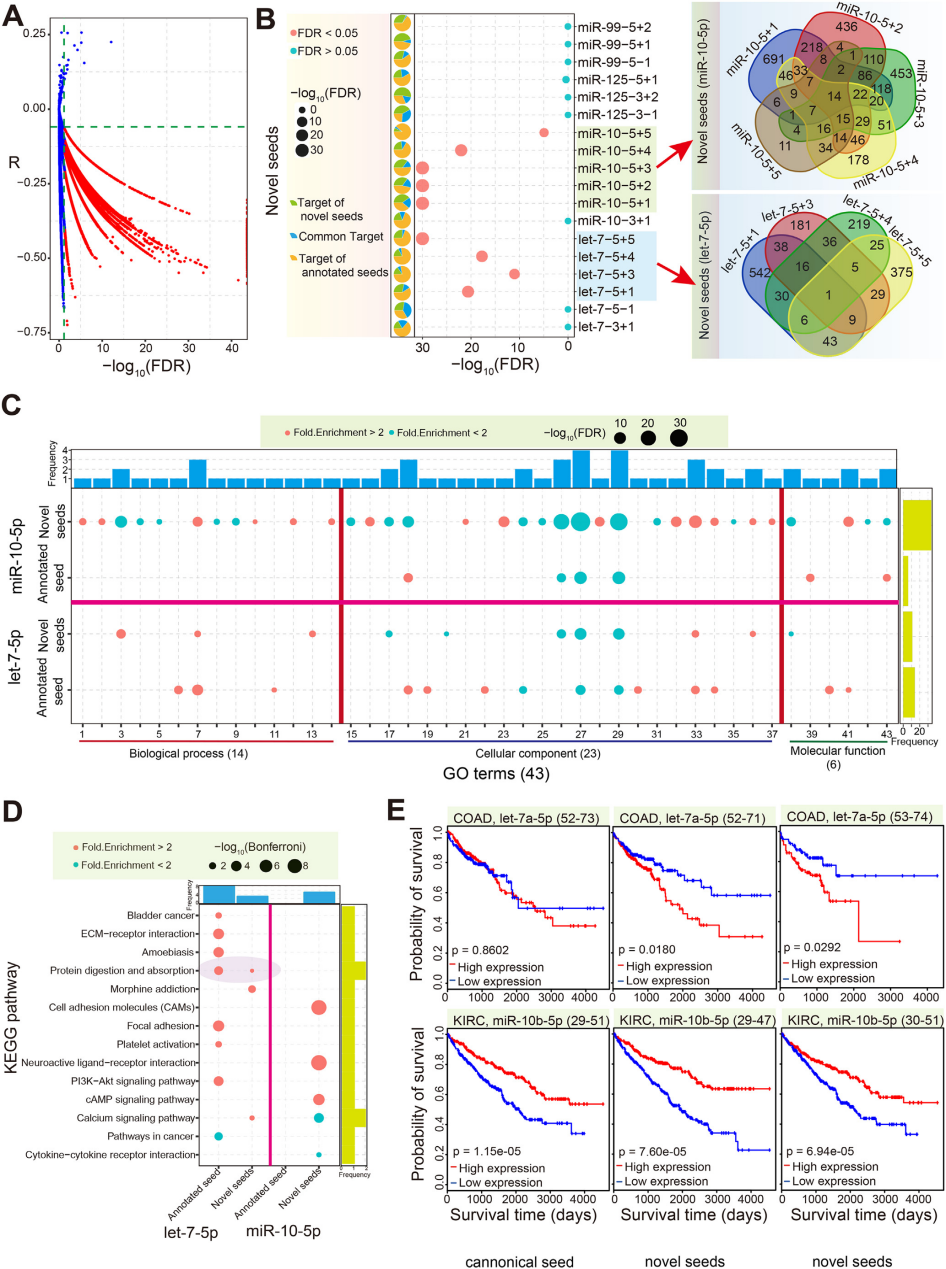
Target validation and analysis of function enrichment. (A) Correlation analysis of isomiRs and their targets. Interactions with a negative correlation (r < 0, FDR < 0.05) are selected for further analysis. (B) Distribution of potential differences between shifted seed sequences and annotated seeds as determined by hypergeometric distribution test, and Venn diagram showing detailed distributions among these novel seeds. (C) Bubble figure showing differences in enriched GO terms between annotated and novel seeds (FDR < 0.05). Biological process (1–14): 1, activation of phospholipase C activity; 2, axon guidance; 3, cell adhesion; 4, cell-cell signaling; 5, chemical synaptic transmission; 6, collagen catabolic process; 7, extracellular matrix organization; 8, homophilic cell adhesion via plasma membrane adhesion molecules; 9, nervous system development; 10, outflow tract morphogenesis; 11, platelet degranulation; 12, positive regulation of angiogenesis; 13, potassium ion transport; 14, smooth muscle contraction. Cellular component (15–37): 15, apical plasma membrane; 16, basolateral plasma membrane; 17, cell junction; 18, cell surface; 19, collagen trimer; 20, dendrite; 21, endocytic vesicle membrane; 22, endoplasmic reticulum lumen; 23, external side of plasma membrane; 24, extracellular region; 25, extracellular space; 26, integral component of membrane; 27, integral component of plasma membrane; 28, neuron projection; 29, plasma membrane; 30, platelet alpha granule lumen; 31, postsynaptic density; 32, postsynaptic membrane; 33, proteinaceous extracellular matrix; 34, receptor complex; 35, synapse; 36, voltage-gated potassium channel complex; 37, Z-disc. Molecular function (38–43): 38, calcium ion binding; 39, cholesterol binding; 40, extracellular matrix structural constituent; 41, heparin binding; 42, receptor activity; 43, transcriptional activator activity, RNA polymerase II core promoter proximal region sequence-specific binding. (D) Bubble figure showing differences in enriched KEGG pathways between annotated and novel seeds (Bonferroni, p < 0.05 and FDR < 0.2). (E) Survival analysis based on different isomiR types.

Based on the predicted target mRNAs of the seed regions of the isomiRs, we further screened candidate targets based on opposite abnormal expression and correlation analysis. A hypergeometric test was then conducted to distinguish potential differences in target mRNAs between annotated seeds and novel seeds. Our results showed that the targets of the novel seeds from let-7-5p and miR-10-5p were significantly different from those of their annotated seeds (FDR < 0.05, Fig. 4A and B). Pairwise comparisons of these novel seeds revealed gain or loss of target mRNAs, although some seed sequence alterations only involved a single nucleotide difference (Fig. 4B). Gain or loss of target mRNAs among novel seeds, and between novel and annotated seeds, may contribute to further functional variation. For example, enriched GO terms and KEGG pathways were inconsistent among the annotated seeds and novel seeds, with many terms or pathways being gained or lost (Fig. 4C and D). Fewer GO terms and KEGG pathways were shared by annotated seeds and novel seeds, with many being specific to either the annotated or novel seeds. Novel seeds in miR-10-5p were associated with a higher number of genes or pathways associated with biological processes compared with the annotated seed through the acquisition of more specific target mRNAs. In comparison, novel seeds in let-7-5p resulted in decreased molecular function through the loss of some targets compared with their annotated seed (Fig. 4C and D). These functional variations strongly support the hypothesis that the previously ignored novel seeds will contribute to novel biological pathways through the gain and/or loss of target mRNAs.

Further analysis of the abnormal isomiRs showed variations in patient survival as a result of the changes, despite some of the isomiRs having identical seed sequences to those of their annotated miRNAs (Fig. 4E), validating the notion that diverse isomiRs should be treated as independent small RNAs. We found that increases and decreases in the expression of annotated let-7a-5p (location 52–73) had no significant impact (p = 0.8602) on the survival of colon adenocarcinoma (COAD) patients. However, significant differences in survival were associated with abnormal let-7a-5p isomiRs with altered chromosomal locations (location 52–71, p = 0.018; location 53–74, p = 0.0292). On the contrary, a more consistent survival trend was observed for miR-10b-5p compared with the annotated and abnormal isomiRs in kidney renal clear cell carcinoma (KIRC) patients (Fig. 4E).

### 3.5 Rewired regulatory networks of novel seeds compared with annotated seeds

**Fig. 5:**
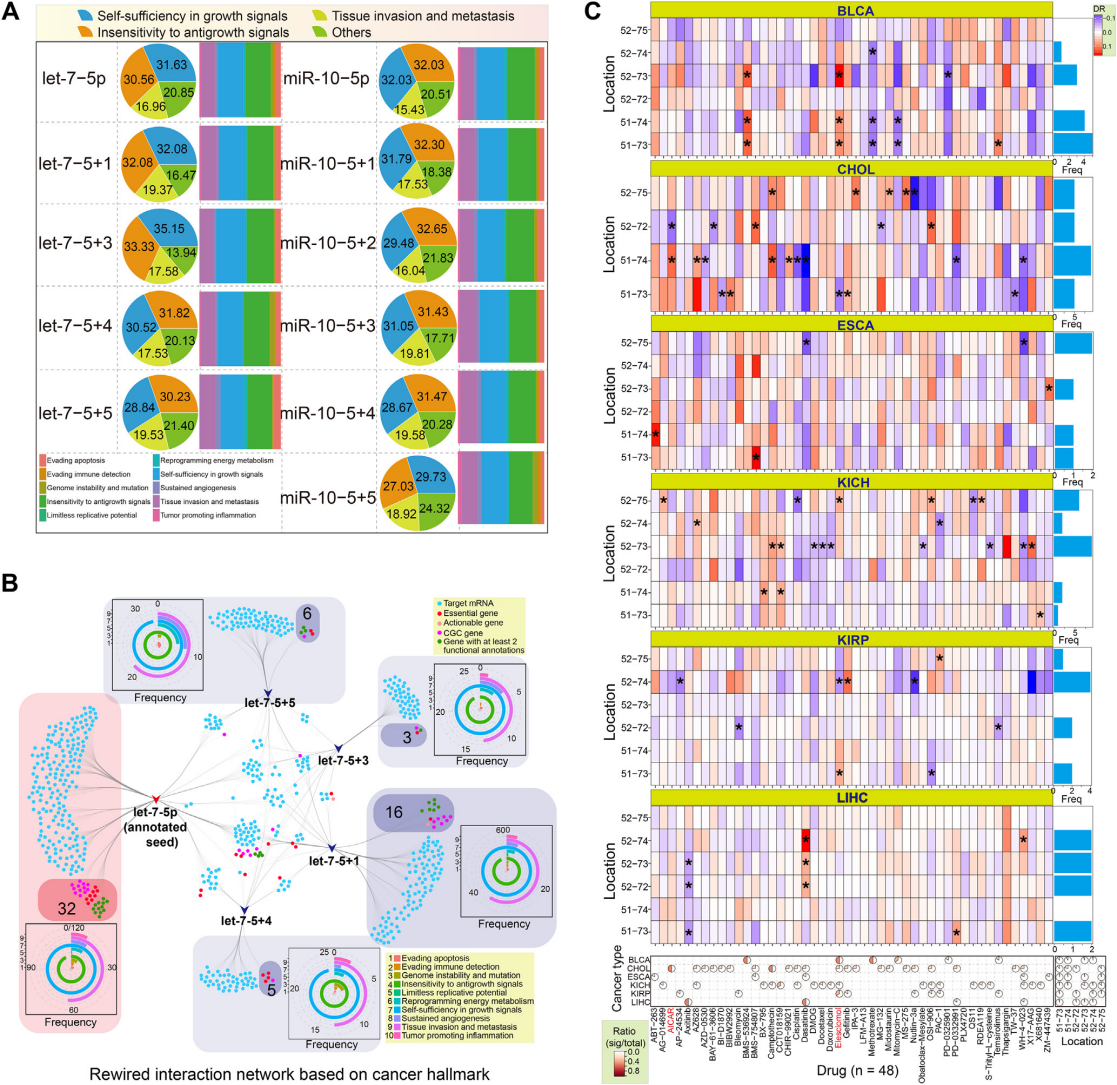
Rewired regulatory networks of novel seeds compared with annotated seeds. (A) Distribution of target genes with functional annotations between novel and annotated seeds, including actionable, CGC, core essential, oncogene, and TSG genes. (B) Rewired isomiR-mRNA interaction network based on target genes associated with cancer hallmarks among seeds from the let-7-5p locus. These genes are further classified according to their functional annotations, including actionable, CGC, core essential, oncogene, and TSG genes. Frequencies of genes associated with cancer hallmarks are also presented for specific targets of each involved seed. (C) Drug responses for six isomiRs (two seeds) of the let-7a-5p locus. A total of 48 drugs are shown, and a significant difference in drug response is observed for each in at least one cancer type. Data for six cancer types are presented (at least five points with significant difference), and other data can be found in Supplementary Fig. S5. Asterisks (*) indicate drugs for which a significant difference was observed between the abnormal expression and normal expression groups (DR > 0.1 or DR <  − 0.1 and p < 0.05). Among the six isomiRs, only four were detected in CHOL. “DR” indicates a difference in the values of the shifted seed group and the annotated seed group. “Freq” indicates frequency. “Sig/total” indicates the ratio of the number of significant results and the total number of isomiRs (pie distribution, left) or the total number of drugs (pie distribution, right). The location annotation provides a detailed locus for each isomiR. For example, 51–73 indicates the location hg38:chr22:46112751–46112773:+.

Compared with canonical miRNAs, isomiRs display diverse sequences, length distributions, and expression patterns, along with potential changes in target mRNAs, all of which may lead to further functional variation. Some specific isomiRs have the potential to be used as diagnostic biomarkers of human disease. Among annotated target genes, including actionable genes, CGC, essential genes, oncogenes, and TSG, we identified obvious differences in the targets of novel seeds and annotated seeds (Supplementary Fig. S4C and S4D). Some target mRNAs were associated with specific cancer hallmarks, and the predominant hallmarks sometimes varied among seeds. However, the top three associated hallmarks were consistent between the two families, and included self-sufficiency in growth signals, tissue invasion and metastasis, and insensitivity to antigrowth signals (Fig. 5A). Because of the common targets associated with cancer hallmarks and genes with special annotations, we also generated interaction networks to show the detailed relationships among targets of different seeds based on cancer hallmarks and KEGG pathways, respectively (Fig. 5B and Supplementary Fig. S4E–G). Analysis of the specific targets of each seed showed an inconsistent number of genes with special annotations, gene distributions of associated cancer hallmarks, and KEGG pathways. For example, the interaction network for let-7-5p based on the cancer hallmarks revealed almost equal numbers of hallmark genes for the annotated and novel seeds. Among the novel seeds of let-7-5p, we found that let-7-5p + 1 had the greatest number of target hallmark genes (*n* = 16) (Fig. 5B). A similar finding was obtained from the rewired interaction network based on the KEGG pathway (Supplementary Fig. S4E).

Finally, to explore the potential differences in drug responses for the isomiRs, we performed an analysis using data from the GDSC database. Based on the expression patterns of the multiple isomiRs of let-7-5p, we selected six isomiRs containing two different seeds for the drug response analysis. Our results confirmed that the various isomiRs induced differing responses across the different cancer types (Fig. 5C and Supplementary Fig. S5). Interestingly, isomiRs that shared the same seed sequence showed diverse drug sensitivities despite only containing alterations at the 3ʹ end of the seed sequence. Further, some isomiRs with different seeds were associated with opposite drug responses. For example, isomiRs located at hg38:chr22:46112751–46112773 (DR = 0.1012, p = 0.0305) and hg38:chr22:46112752–46112774 (DR =  − 0.1112, p = 0.0277) caused opposing responses to Elesclomol in KIRP patients, while hg38:chr22:46112751–46112774 (DR = 0.2302, p = 0.0163) and hg38:chr22:46112752–46112772 (DR =  − 0.1890, p = 0.0313) caused opposite responses to AICAR drug in CHOL patients (Fig. 5C).

## 4 Discussion

This study indicates that the production of multiple highly similar isomiRs is a standard outcome of the evolutionary process. We examined the repertoire of small RNAs belonging to the let-7 and miR-10 gene families, which contain multiple isomiRs with length, sequence, and expression heterogeneities, and assessed potential associations among homologous and/or clustered miRNA loci. The various isomiRs generated from a single miRNA locus in a specific species is a miniature of canonical miRNA repertoires across animal species, and the presence of multiple sequences allows the flexibility to select optimal regulatory molecules. Decreased expression of isomiRs from the let-7 and miR-10 gene families was observed in various cancers (Fig. 1A). This is consistent with previous experimental [44]. The expression of let-7 and miR-10 miRNAs was regulated at various stages during biogenesis, and also depended on the cell type. Many factors controlled the dynamic expression. With a focus on BRCA, we searched for TFs that regulated let-7 and miR-10 members via promoter interactions using TransmiR version 2.0. The expression of some TFs, including EGR1, IL-6, EBF1, and FOXO1, was positively correlated with the expression of isomiRs in BRCA patients. However, other highly expressed TFs, such as APOBEC3B, EZH2, E2F1, GATA3, and MYB, were negatively correlated with isomiRs from the two selected gene families. Therefore, these dysregulated TFs likely contribute to the reduced expression of isomiRs.

Numerous oncogenes and signaling pathways were demonstrated to be targets of let-7 and miR-10 miRNAs in the current study. A number of groups have reported that miRNA variants involved in regulating distinctive target genes play an important biological role in controlling miRNA-mediated gene expression [3], [45], [46]. Tan et al. used luciferase assays to confirm predicted differences between miRNA and isomiR pairs in three out of six cases (including PTEN, CDH1, DNMT3B, NCAM2, HMGA2, and BTG1)[47]. Ma et al. showed that the expression of metabolism-related proteins, including PEPCK, G6Pase, FAS, and CPT1A, was significantly suppressed by canonical miR-27b. In contrast, the expression of these proteins was only slightly inhibited by isomiR-27b-1 or isomiR-27b-2 following transfection into AML-12 cells [48]. It is likely that comprehensive analysis would reveal that this is not a rare phenomenon. Thus, variations in the expression of multiple isomiRs and cross-talk among the isomiRs provide hidden information for the coding-non-coding RNA interaction networks. Our study confirms that isomiRs with novel seeds disturb coding-non-coding RNA regulatory networks and pathways and lead to functional alterations through the gain and/or loss of target mRNAs. Altered interactions and rewired networks strongly support the hypothesis that isomiRs will result in functional variation.

Further, the observed differences in survival and drug response among the different isomiRs confirm that the various isomiRs should not be ignored. The isomiRs of let-7a-5p may have different clinical relevance in terms of survival despite having the same functional regions (Fig. 4E). The coding-non-coding RNA regulatory network based on cancer hallmark genes (Fig. 5B) indicate a novel and more complex network than the single network considered the canonical miRNA only. This is because novel seeds are involved in the coding-non-coding network and altered the original interactions. The rewiring network enrich and complicate the coding-non-coding RNA regulatory network at the isomiR level by adding more seeds and additional target mRNAs. The isomiRs also have the differences in analysis of drug responses (Fig. 5C and Fig. S5), and isomiRs with novel seeds may cause opposite results despite being generated from the same miRNA locus and showing highly similar sequence relationships. Similar results could be extended to multiple isomiRs from homologous miRNAs and canonical miRNAs, although homologous miRNAs are usually studied together because they often have the same functional regions. IsomiRs containing sequence variations may have additional biological functions that should be the focus of further studies. These observations support the hypothesis that isomiRs exhibiting sequence divergence are functional regulatory molecules, and that they may contribute to biological processes via coordinated interactions in regulatory networks. Changes at the miRNA isoform level likely occur in tandem with disease progression, meaning that further attention should be given to cancer-related miRNAs not only at the classical miRNA expression level, but also at the isoform level, when considering disease-ameliorating therapies.

In summary, this study identifies a series of isomiRs of the let-7 and miR-10 gene families that display significant differences from their annotated miRNAs. Our findings also confirm that the isomiR repertoire is closely associated with homologous and/or clustered miRNA genes. IsomiRs with different sequences may have diverse clinical relevance, indicating their potential diagnostic role. Further studies should examine nucleotide variation in small RNAs, and focus on the biological functions of the isomiRs using experimental validation.

## Funding

This work was supported by the 10.13039/501100001809National Natural Science Foundation of China [grant number 61771251]; the Key Project of Social Development in Jiangsu Province [grant number BE2016773]; the National Natural Science Foundation of Jiangsu [grant number BK20171443]; the Qinglan Project in Jiangsu Province; Nanjing Normal University Overseas Studies [grant number NJNU-2017]; NUPTSF [grant numbers NY220041]; and the Priority Academic Program Development of Jiangsu Higher Education Institutions (PAPD).

## Authorship contribution statement

**Tingming Liang:** Conceptualization, Visualization, Methodology, Software, Writing - original draft. **Leng Han:** Data curation, Software, Supervision. **Li Guo:** Conceptualization, Visualization, Investigation, Supervision, Writing - original draft.

## Declaration of Competing Interest

The authors declare that they have no known competing financial interests or personal relationships that could have appeared to influence the work reported in this paper.

## References

[1] Guo H., Ingolia N.T., Weissman J.S., Bartel D.P. (2010). Mammalian microRNAs predominantly act to decrease target mRNA levels. Nature.

[2] Krol J., Loedige I., Filipowicz W. (2010). The widespread regulation of microRNA biogenesis, function and decay. Nat Rev Genet.

[3] Ameres S.L., Zamore P.D. (2013). Diversifying microRNA sequence and function. Nat Rev Mol Cell Biol.

[4] Shukla G.C., Singh J., Barik S. (2011). MicroRNAs: processing, maturation, target recognition and regulatory functions. Mol Cell Pharmacol.

[5] Bartel D.P. (2009). MicroRNAs: target recognition and regulatory functions. Cell.

[6] Neilsen C.T., Goodall G.J., Bracken C.P. (2012). IsomiRs–the overlooked repertoire in the dynamic microRNAome. Trends Genet.

[7] Brueckner B., Stresemann C., Kuner R., Mund C., Musch T. (2007). The human let-7a-3 locus contains an epigenetically regulated microRNA gene with oncogenic function. Cancer Res.

[8] Kuppusamy K.T., Jones D.C., Sperber H., Madan A., Fischer K.A. (2015). Let-7 family of microRNA is required for maturation and adult-like metabolism in stem cell-derived cardiomyocytes. Proc Natl Acad Sci USA.

[9] Tan W., Li Y., Lim S.G., Tan T.M. (2014). miR-106b-25/miR-17-92 clusters: polycistrons with oncogenic roles in hepatocellular carcinoma. World J Gastroenterol.

[10] Chen J., Huang Z.P., Seok H.Y., Ding J., Kataoka M. (2013). mir-17-92 cluster is required for and sufficient to induce cardiomyocyte proliferation in postnatal and adult hearts. Circ Res.

[11] Salem O., Erdem N., Jung J., Münstermann E., Wörner A. (2016). The highly expressed 5’isomiR of hsa-miR-140-3p contributes to the tumor-suppressive effects of miR-140 by reducing breast cancer proliferation and migration. BMC Genomics.

[12] Bhardwaj A., Singh H., Trinidad C.M., Albarracin C.T., Hunt K.K. (2018). The isomiR-140-3p-regulated mevalonic acid pathway as a potential target for prevention of triple negative breast cancer. Breast Cancer Res.

[13] Telonis A.G., Magee R., Loher P., Chervoneva I., Londin E. (2017). Knowledge about the presence or absence of miRNA isoforms (isomiRs) can successfully discriminate amongst 32 TCGA cancer types. Nucleic Acids Res.

[14] Telonis A.G., Loher P., Jing Y., Londin E., Rigoutsos I. (2015). Beyond the one-locus-one-miRNA paradigm: microRNA isoforms enable deeper insights into breast cancer heterogeneity. Nucleic Acids Res.

[15] Loher P., Londin E.R., Rigoutsos I. (2014). IsomiR expression profiles in human lymphoblastoid cell lines exhibit population and gender dependencies. Oncotarget.

[16] Londin E., Magee R., Shields C.L., Lally S.E., Sato T. (2019). IsomiRs and tRNA-derived fragments are associated with metastasis and patient survival in uveal melanoma. Pigm Cell Melanoma R.

[17] Reinhart B.J., Slack F.J., Basson M., Pasquinelli A.E., Bettinger J.C. (2000). The 21-nucleotide let-7 RNA regulates developmental timing in Caenorhabditis elegans. Nature.

[18] Jiang X., Hawkins J.S., Lee J., Lizama C.O., Bos F.L. (2017). Let-7 microRNA-dependent control of leukotriene signaling regulates the transition of hematopoietic niche in mice. Nat Commun.

[19] Yu F., Yao H., Zhu P., Zhang X., Pan Q. (2007). let-7 regulates self renewal and tumorigenicity of breast cancer cells. Cell.

[20] Takamizawa J., Konishi H., Yanagisawa K., Tomida S., Osada H. (2004). Reduced expression of the let-7 microRNAs in human lung cancers in association with shortened postoperative survival. Cancer Res.

[21] Worringer K.A., Rand T.A., Hayashi Y., Sami S., Takahashi K. (2014). The let-7/LIN-41 pathway regulates reprogramming to human induced pluripotent stem cells by controlling expression of prodifferentiation genes. Cell Stem Cell.

[22] Manier S., Powers J.T., Sacco A., Glavey S.V., Huynh D. (2017). The LIN28B/let-7 axis is a novel therapeutic pathway in multiple myeloma. Leukemia.

[23] Yan L., Zhou J., Gao Y., Ghazal S., Lu L. (2015). Regulation of tumor cell migration and invasion by the H19/let-7 axis is antagonized by metformin-induced DNA methylation. Oncogene.

[24] Kozomara A., Birgaoanu M., Griffiths-Jones S. (2019). miRBase: from microRNA sequences to function. Nucleic Acids Res.

[25] Colaprico A., Silva T.C., Olsen C., Garofano L., Cava C. (2016). TCGAbiolinks: an R/Bioconductor package for integrative analysis of TCGA data. Nucleic Acids Res.

[26] Subramanian A., Tamayo P., Mootha V.K., Mukherjee S., Ebert B.L. (2005). Gene set enrichment analysis: a knowledge-based approach for interpreting genome-wide expression profiles. Proc Natl Acad Sci USA.

[27] Futreal P.A., Coin L., Marshall M., Down T., Hubbard T. (2004). A census of human cancer genes. Nat Rev Cancer.

[28] Hart T., Chandrashekhar M., Aregger M., Steinhart Z., Brown K.R. (2015). High-resolution CRISPR screens reveal fitness genes and genotype-specific cancer liabilities. Cell.

[29] Blomen V.A., Majek P., Jae L.T., Bigenzahn J.W., Nieuwenhuis J. (2015). Gene essentiality and synthetic lethality in haploid human cells. Science.

[30] Wang T., Birsoy K., Hughes N.W., Krupczak K.M., Post Y. (2015). Identification and characterization of essential genes in the human genome. Science.

[31] Vogelstein B., Papadopoulos N., Velculescu V.E., Zhou S., Diaz L.A. (2013). Cancer genome landscapes. Science.

[32] Tong Z., Cui Q., Wang J., Zhou Y. (2019). TransmiR v2. 0: an updated transcription factor-microRNA regulation database. Nucleic Acids Res.

[33] Orostica K.Y., Verdugo R.A. (2016). chromPlot: visualization of genomic data in chromosomal context. Bioinformatics.

[34] Larkin M.A., Blackshields G., Brown N.P., Chenna R., Mcgettigan P.A. (2007). Clustal w and clustal x version 2.0. Bioinformatics.

[35] Kumar S., Stecher G., Tamura K. (2016). MEGA7: molecular evolutionary genetics analysis version 7.0 for bigger datasets. Mol Biol Evol.

[36] Huson D.H. (1998). SplitsTree: analyzing and visualizing evolutionary data. Bioinformatics.

[37] Crooks G.E., Hon G., Chandonia J.M., Brenner S.E. (2004). WebLogo: a sequence logo generator. Genome Res.

[38] Love M.I., Huber W., Anders S. (2014). Moderated estimation of fold change and dispersion for RNA-seq data with DESeq2. Genome Biol.

[39] Agarwal V., Bell G.W., Nam J.W., Bartel D.P. (2015). Predicting effective microRNA target sites in mammalian mRNAs. Elife.

[40] Da W.H., Sherman B.T., Lempicki R.A. (2008). Systematic and integrative analysis of large gene lists using DAVID bioinformatics resources. Nature Protoc.

[41] Subramanian A., Tamayo P., Mootha V.K., Mukherjee S., Ebert B.L. (2005). Gene set enrichment analysis: a knowledge-based approach for interpreting genome-wide expression profiles. Proc Natl Acad Sci USA.

[42] Shannon P., Markiel A., Ozier O., Baliga N.S., Wang J.T. (2003). Cytoscape: a software environment for integrated models of biomolecular interaction networks. Genome Res.

[43] Yang W., Soares J., Greninger P., Edelman E.J., Lightfoot H. (2013). Genomics of Drug Sensitivity in Cancer (GDSC): a resource for therapeutic biomarker discovery in cancer cells. Nucleic Acids Res.

[44] Schultz J., Lorenz P., Gross G., Ibrahim S., Kunz M. (2008). MicroRNA let-7b targets important cell cycle molecules in malignant melanoma cells and interferes with anchorage-independent growth. Cell Res.

[45] Hinton A., Hunter S.E., Afrikanova I., Jones G.A., Lopez A.D. (2014). sRNA-seq analysis of human embryonic stem cells and definitive endoderm reveals differentially expressed microRNAs and novel IsomiRs with distinct targets. Stem Cells.

[46] Vickers K.C., Sethupathy P., Baran-Gale J., Remaley A.T. (2013). Complexity of microRNA function and the role of isomiRs in lipid homeostasis. J Lipid Res.

[47] Tan G.C., Chan E., Molnar A., Sarkar R., Alexieva D. (2014). 5' isomiR variation is of functional and evolutionary importance. Nucleic Acids Res.

[48] Ma M., Yin Z., Zhong H., Liang T., Guo L. (2019). Analysis of the expression, function, and evolution of miR-27 isoforms and their responses in metabolic processes. Genomics.

