# Supplementary material for: Rewired functional regulatory networks among miRNA isoforms (isomiRs) from let-7 and miR-10 gene families in cancer
Source: Comput Struct Biotechnol J. 2020 May 13;18:1238–48. doi: 10.1016/j.csbj.2020.05.001 (PMC7280754; doi:10.1016/j.csbj.2020.05.001)
Supplement: Supplementary data 1 [file mmc1.pdf]

Table S1

Cancer	isomiR	Freq	Group
BLCA	hsa-let-7a-1&mature,MIMAT0000062	5	tumor
BLCA	hsa-let-7a-1&mature,MIMAT0004481	1	tumor
BLCA	hsa-let-7a-2&mature,MIMAT0000062	5	tumor
BLCA	hsa-let-7a-2&mature,MIMAT0010195	1	tumor
BLCA	hsa-let-7a-3&mature,MIMAT0000062	9	tumor
BLCA	hsa-let-7a-3&mature,MIMAT0004481	1	tumor
BLCA	hsa-let-7b&mature,MIMAT0000063	8	tumor
BLCA	hsa-let-7b&mature,MIMAT0004482	1	tumor
BLCA	hsa-let-7c&mature,MIMAT0000064	9	tumor
BLCA	hsa-let-7c&mature,MIMAT0026472	1	tumor
BLCA	hsa-let-7d&mature,MIMAT0000065	4	tumor
BLCA	hsa-let-7d&mature,MIMAT0004484	4	tumor
BLCA	hsa-let-7e&mature,MIMAT0000066	6	tumor
BLCA	hsa-let-7e&mature,MIMAT0004485	1	tumor
BLCA	hsa-let-7f-1&mature,MIMAT0000067	5	tumor
BLCA	hsa-let-7f-2&mature,MIMAT0000067	6	tumor
BLCA	hsa-let-7g&mature,MIMAT0000414	4	tumor
BLCA	hsa-let-7g&mature,MIMAT0004584	1	tumor
BLCA	hsa-let-7i&mature,MIMAT0000415	3	tumor
BLCA	hsa-let-7i&mature,MIMAT0004585	3	tumor
BLCA	hsa-mir-100&mature,MIMAT0000098	8	tumor
BLCA	hsa-mir-10a&mature,MIMAT0000253	26	tumor
BLCA	hsa-mir-10b&mature,MIMAT0000254	20	tumor
BLCA	hsa-mir-10b&mature,MIMAT0004556	1	tumor
BLCA	hsa-mir-125a&mature,MIMAT0000443	4	tumor
BLCA	hsa-mir-125b-1&mature,MIMAT0000423	3	tumor
BLCA	hsa-mir-125b-2&mature,MIMAT0000423	2	tumor
BLCA	hsa-mir-125b-2&mature,MIMAT0004603	7	tumor
BLCA	hsa-mir-98&mature,MIMAT0000096	3	tumor
BLCA	hsa-mir-99a&mature,MIMAT0000097	6	tumor
BLCA	hsa-mir-99b&mature,MIMAT0000689	10	tumor
BLCA	hsa-mir-99b&mature,MIMAT0004678	5	tumor
BLCA	hsa-let-7a-1&mature,MIMAT0000062	5	normal
BLCA	hsa-let-7a-2&mature,MIMAT0000062	5	normal
BLCA	hsa-let-7a-3&mature,MIMAT0000062	5	normal
BLCA	hsa-let-7b&mature,MIMAT0000063	5	normal
BLCA	hsa-let-7c&mature,MIMAT0000064	5	normal
BLCA	hsa-let-7d&mature,MIMAT0000065	3	normal
BLCA	hsa-let-7d&mature,MIMAT0004484	2	normal
BLCA	hsa-let-7e&mature,MIMAT0000066	3	normal
BLCA	hsa-let-7f-1&mature,MIMAT0000067	3	normal

BLCA	hsa-let-7f-2&mature,MIMAT0000067	5	normal
BLCA	hsa-let-7g&mature,MIMAT0000414	2	normal
BLCA	hsa-let-7i&mature,MIMAT0000415	3	normal
BLCA	hsa-mir-100&mature,MIMAT0000098	5	normal
BLCA	hsa-mir-10a&mature,MIMAT0000253	11	normal
BLCA	hsa-mir-10b&mature,MIMAT0000254	13	normal
BLCA	hsa-mir-125a&mature,MIMAT0000443	4	normal
BLCA	hsa-mir-125b-1&mature,MIMAT0000423	2	normal
BLCA	hsa-mir-125b-2&mature,MIMAT0000423	2	normal
BLCA	hsa-mir-99a&mature,MIMAT0000097	3	normal
BLCA	hsa-mir-99b&mature,MIMAT0000689	5	normal
BRCA	hsa-let-7a-1&mature,MIMAT0000062	9	tumor
BRCA	hsa-let-7a-1&mature,MIMAT0004481	2	tumor
BRCA	hsa-let-7a-2&mature,MIMAT0000062	9	tumor
BRCA	hsa-let-7a-2&mature,MIMAT0010195	1	tumor
BRCA	hsa-let-7a-3&mature,MIMAT0000062	11	tumor
BRCA	hsa-let-7a-3&mature,MIMAT0004481	2	tumor
BRCA	hsa-let-7b&mature,MIMAT0000063	17	tumor
BRCA	hsa-let-7b&mature,MIMAT0004482	2	tumor
BRCA	hsa-let-7c&mature,MIMAT0000064	11	tumor
BRCA	hsa-let-7c&mature,MIMAT0026472	2	tumor
BRCA	hsa-let-7d&mature,MIMAT0000065	5	tumor
BRCA	hsa-let-7d&mature,MIMAT0004484	4	tumor
BRCA	hsa-let-7e&mature,MIMAT0000066	4	tumor
BRCA	hsa-let-7e&mature,MIMAT0004485	1	tumor
BRCA	hsa-let-7f-1&mature,MIMAT0000067	8	tumor
BRCA	hsa-let-7f-2&mature,MIMAT0000067	7	tumor
BRCA	hsa-let-7g&mature,MIMAT0000414	7	tumor
BRCA	hsa-let-7g&mature,MIMAT0004584	1	tumor
BRCA	hsa-let-7i&mature,MIMAT0000415	4	tumor
BRCA	hsa-let-7i&mature,MIMAT0004585	3	tumor
BRCA	hsa-mir-100&mature,MIMAT0000098	9	tumor
BRCA	hsa-mir-10a&mature,MIMAT0000253	30	tumor
BRCA	hsa-mir-10b&mature,MIMAT0000254	23	tumor
BRCA	hsa-mir-10b&mature,MIMAT0004556	1	tumor
BRCA	hsa-mir-125a&mature,MIMAT0000443	5	tumor
BRCA	hsa-mir-125b-1&mature,MIMAT0000423	4	tumor
BRCA	hsa-mir-125b-2&mature,MIMAT0000423	4	tumor
BRCA	hsa-mir-125b-2&mature,MIMAT0004603	4	tumor
BRCA	hsa-mir-98&mature,MIMAT0000096	3	tumor
BRCA	hsa-mir-99a&mature,MIMAT0000097	9	tumor
BRCA	hsa-mir-99b&mature,MIMAT0000689	12	tumor
BRCA	hsa-mir-99b&mature,MIMAT0004678	5	tumor
BRCA	hsa-let-7a-1&mature,MIMAT0000062	5	normal

BRCA	hsa-let-7a-2&mature,MIMAT0000062	5	normal
BRCA	hsa-let-7a-3&mature,MIMAT0000062	6	normal
BRCA	hsa-let-7b&mature,MIMAT0000063	8	normal
BRCA	hsa-let-7b&mature,MIMAT0004482	1	normal
BRCA	hsa-let-7c&mature,MIMAT0000064	7	normal
BRCA	hsa-let-7c&mature,MIMAT0026472	1	normal
BRCA	hsa-let-7d&mature,MIMAT0000065	3	normal
BRCA	hsa-let-7d&mature,MIMAT0004484	4	normal
BRCA	hsa-let-7e&mature,MIMAT0000066	4	normal
BRCA	hsa-let-7f-1&mature,MIMAT0000067	5	normal
BRCA	hsa-let-7f-2&mature,MIMAT0000067	5	normal
BRCA	hsa-let-7g&mature,MIMAT0000414	3	normal
BRCA	hsa-let-7g&mature,MIMAT0004584	1	normal
BRCA	hsa-let-7i&mature,MIMAT0000415	3	normal
BRCA	hsa-let-7i&mature,MIMAT0004585	3	normal
BRCA	hsa-mir-100&mature,MIMAT0000098	8	normal
BRCA	hsa-mir-10a&mature,MIMAT0000253	16	normal
BRCA	hsa-mir-10b&mature,MIMAT0000254	21	normal
BRCA	hsa-mir-10b&mature,MIMAT0004556	1	normal
BRCA	hsa-mir-125a&mature,MIMAT0000443	5	normal
BRCA	hsa-mir-125b-1&mature,MIMAT0000423	4	normal
BRCA	hsa-mir-125b-2&mature,MIMAT0000423	4	normal
BRCA	hsa-mir-125b-2&mature,MIMAT0004603	1	normal
BRCA	hsa-mir-99a&mature,MIMAT0000097	9	normal
BRCA	hsa-mir-99b&mature,MIMAT0000689	9	normal
BRCA	hsa-mir-99b&mature,MIMAT0004678	1	normal
CHOL	hsa-let-7a-1&mature,MIMAT0000062	5	tumor
CHOL	hsa-let-7a-2&mature,MIMAT0000062	5	tumor
CHOL	hsa-let-7a-3&mature,MIMAT0000062	5	tumor
CHOL	hsa-let-7b&mature,MIMAT0000063	6	tumor
CHOL	hsa-let-7c&mature,MIMAT0000064	5	tumor
CHOL	hsa-let-7d&mature,MIMAT0000065	3	tumor
CHOL	hsa-let-7d&mature,MIMAT0004484	3	tumor
CHOL	hsa-let-7e&mature,MIMAT0000066	3	tumor
CHOL	hsa-let-7f-1&mature,MIMAT0000067	3	tumor
CHOL	hsa-let-7f-2&mature,MIMAT0000067	4	tumor
CHOL	hsa-let-7g&mature,MIMAT0000414	3	tumor
CHOL	hsa-let-7g&mature,MIMAT0004584	1	tumor
CHOL	hsa-let-7i&mature,MIMAT0000415	3	tumor
CHOL	hsa-let-7i&mature,MIMAT0004585	2	tumor
CHOL	hsa-mir-100&mature,MIMAT0000098	6	tumor
CHOL	hsa-mir-10a&mature,MIMAT0000253	14	tumor
CHOL	hsa-mir-10b&mature,MIMAT0000254	10	tumor
CHOL	hsa-mir-125a&mature,MIMAT0000443	4	tumor

CHOL	hsa-mir-125b-1&mature,MIMAT0000423	2	tumor
CHOL	hsa-mir-125b-2&mature,MIMAT0000423	2	tumor
CHOL	hsa-mir-98&mature,MIMAT0000096	1	tumor
CHOL	hsa-mir-99a&mature,MIMAT0000097	2	tumor
CHOL	hsa-mir-99b&mature,MIMAT0000689	5	tumor
CHOL	hsa-mir-99b&mature,MIMAT0004678	1	tumor
CHOL	hsa-let-7a-1&mature,MIMAT0000062	5	normal
CHOL	hsa-let-7a-2&mature,MIMAT0000062	5	normal
CHOL	hsa-let-7a-3&mature,MIMAT0000062	5	normal
CHOL	hsa-let-7b&mature,MIMAT0000063	5	normal
CHOL	hsa-let-7c&mature,MIMAT0000064	5	normal
CHOL	hsa-let-7d&mature,MIMAT0000065	1	normal
CHOL	hsa-let-7d&mature,MIMAT0004484	2	normal
CHOL	hsa-let-7e&mature,MIMAT0000066	3	normal
CHOL	hsa-let-7f-1&mature,MIMAT0000067	3	normal
CHOL	hsa-let-7f-2&mature,MIMAT0000067	4	normal
CHOL	hsa-let-7g&mature,MIMAT0000414	3	normal
CHOL	hsa-let-7i&mature,MIMAT0000415	3	normal
CHOL	hsa-mir-100&mature,MIMAT0000098	4	normal
CHOL	hsa-mir-10a&mature,MIMAT0000253	11	normal
CHOL	hsa-mir-10b&mature,MIMAT0000254	5	normal
CHOL	hsa-mir-125a&mature,MIMAT0000443	3	normal
CHOL	hsa-mir-125b-1&mature,MIMAT0000423	2	normal
CHOL	hsa-mir-125b-2&mature,MIMAT0000423	2	normal
CHOL	hsa-mir-99a&mature,MIMAT0000097	4	normal
CHOL	hsa-mir-99b&mature,MIMAT0000689	3	normal
COAD	hsa-let-7a-1&mature,MIMAT0000062	7	tumor
COAD	hsa-let-7a-2&mature,MIMAT0000062	7	tumor
COAD	hsa-let-7a-3&mature,MIMAT0000062	7	tumor
COAD	hsa-let-7b&mature,MIMAT0000063	9	tumor
COAD	hsa-let-7b&mature,MIMAT0004482	1	tumor
COAD	hsa-let-7c&mature,MIMAT0000064	5	tumor
COAD	hsa-let-7c&mature,MIMAT0026472	1	tumor
COAD	hsa-let-7d&mature,MIMAT0000065	3	tumor
COAD	hsa-let-7d&mature,MIMAT0004484	4	tumor
COAD	hsa-let-7e&mature,MIMAT0000066	4	tumor
COAD	hsa-let-7e&mature,MIMAT0004485	1	tumor
COAD	hsa-let-7f-1&mature,MIMAT0000067	6	tumor
COAD	hsa-let-7f-2&mature,MIMAT0000067	7	tumor
COAD	hsa-let-7g&mature,MIMAT0000414	4	tumor
COAD	hsa-let-7g&mature,MIMAT0004584	1	tumor
COAD	hsa-let-7i&mature,MIMAT0000415	3	tumor
COAD	hsa-let-7i&mature,MIMAT0004585	3	tumor
COAD	hsa-mir-100&mature,MIMAT0000098	7	tumor

COAD	hsa-mir-10a&mature,MIMAT0000253	31	tumor
COAD	hsa-mir-10b&mature,MIMAT0000254	25	tumor
COAD	hsa-mir-125a&mature,MIMAT0000443	5	tumor
COAD	hsa-mir-125b-1&mature,MIMAT0000423	3	tumor
COAD	hsa-mir-125b-2&mature,MIMAT0000423	3	tumor
COAD	hsa-mir-98&mature,MIMAT0000096	2	tumor
COAD	hsa-mir-99a&mature,MIMAT0000097	3	tumor
COAD	hsa-mir-99b&mature,MIMAT0000689	10	tumor
COAD	hsa-mir-99b&mature,MIMAT0004678	4	tumor
COAD	hsa-let-7a-1&mature,MIMAT0000062	5	normal
COAD	hsa-let-7a-2&mature,MIMAT0000062	5	normal
COAD	hsa-let-7a-3&mature,MIMAT0000062	5	normal
COAD	hsa-let-7b&mature,MIMAT0000063	8	normal
COAD	hsa-let-7b&mature,MIMAT0004482	1	normal
COAD	hsa-let-7c&mature,MIMAT0000064	5	normal
COAD	hsa-let-7d&mature,MIMAT0000065	3	normal
COAD	hsa-let-7d&mature,MIMAT0004484	7	normal
COAD	hsa-let-7e&mature,MIMAT0000066	3	normal
COAD	hsa-let-7e&mature,MIMAT0004485	1	normal
COAD	hsa-let-7f-1&mature,MIMAT0000067	3	normal
COAD	hsa-let-7f-2&mature,MIMAT0000067	3	normal
COAD	hsa-let-7g&mature,MIMAT0000414	2	normal
COAD	hsa-let-7i&mature,MIMAT0000415	3	normal
COAD	hsa-let-7i&mature,MIMAT0004585	1	normal
COAD	hsa-mir-100&mature,MIMAT0000098	2	normal
COAD	hsa-mir-10a&mature,MIMAT0000253	10	normal
COAD	hsa-mir-10b&mature,MIMAT0000254	11	normal
COAD	hsa-mir-125a&mature,MIMAT0000443	5	normal
COAD	hsa-mir-125b-1&mature,MIMAT0000423	3	normal
COAD	hsa-mir-125b-2&mature,MIMAT0000423	3	normal
COAD	hsa-mir-99a&mature,MIMAT0000097	2	normal
COAD	hsa-mir-99b&mature,MIMAT0000689	8	normal
COAD	hsa-mir-99b&mature,MIMAT0004678	1	normal
ESCA	hsa-let-7a-1&mature,MIMAT0000062	5	tumor
ESCA	hsa-let-7a-2&mature,MIMAT0000062	5	tumor
ESCA	hsa-let-7a-3&mature,MIMAT0000062	7	tumor
ESCA	hsa-let-7b&mature,MIMAT0000063	5	tumor
ESCA	hsa-let-7c&mature,MIMAT0000064	7	tumor
ESCA	hsa-let-7c&mature,MIMAT0026472	1	tumor
ESCA	hsa-let-7d&mature,MIMAT0000065	4	tumor
ESCA	hsa-let-7d&mature,MIMAT0004484	3	tumor
ESCA	hsa-let-7e&mature,MIMAT0000066	5	tumor
ESCA	hsa-let-7f-1&mature,MIMAT0000067	5	tumor
ESCA	hsa-let-7f-2&mature,MIMAT0000067	5	tumor

ESCA	hsa-let-7g&mature,MIMAT0000414	4	tumor
ESCA	hsa-let-7g&mature,MIMAT0004584	1	tumor
ESCA	hsa-let-7i&mature,MIMAT0000415	3	tumor
ESCA	hsa-let-7i&mature,MIMAT0004585	2	tumor
ESCA	hsa-mir-100&mature,MIMAT0000098	5	tumor
ESCA	hsa-mir-10a&mature,MIMAT0000253	14	tumor
ESCA	hsa-mir-10b&mature,MIMAT0000254	10	tumor
ESCA	hsa-mir-125a&mature,MIMAT0000443	4	tumor
ESCA	hsa-mir-125b-1&mature,MIMAT0000423	2	tumor
ESCA	hsa-mir-125b-2&mature,MIMAT0000423	2	tumor
ESCA	hsa-mir-125b-2&mature,MIMAT0004603	3	tumor
ESCA	hsa-mir-98&mature,MIMAT0000096	3	tumor
ESCA	hsa-mir-99a&mature,MIMAT0000097	4	tumor
ESCA	hsa-mir-99b&mature,MIMAT0000689	6	tumor
ESCA	hsa-mir-99b&mature,MIMAT0004678	1	tumor
ESCA	hsa-let-7a-1&mature,MIMAT0000062	5	normal
ESCA	hsa-let-7a-2&mature,MIMAT0000062	5	normal
ESCA	hsa-let-7a-3&mature,MIMAT0000062	5	normal
ESCA	hsa-let-7b&mature,MIMAT0000063	5	normal
ESCA	hsa-let-7b&mature,MIMAT0004482	1	normal
ESCA	hsa-let-7c&mature,MIMAT0000064	5	normal
ESCA	hsa-let-7d&mature,MIMAT0000065	2	normal
ESCA	hsa-let-7d&mature,MIMAT0004484	2	normal
ESCA	hsa-let-7e&mature,MIMAT0000066	4	normal
ESCA	hsa-let-7f-1&mature,MIMAT0000067	4	normal
ESCA	hsa-let-7f-2&mature,MIMAT0000067	4	normal
ESCA	hsa-let-7g&mature,MIMAT0000414	2	normal
ESCA	hsa-let-7i&mature,MIMAT0000415	3	normal
ESCA	hsa-mir-100&mature,MIMAT0000098	3	normal
ESCA	hsa-mir-10a&mature,MIMAT0000253	12	normal
ESCA	hsa-mir-10b&mature,MIMAT0000254	9	normal
ESCA	hsa-mir-125a&mature,MIMAT0000443	4	normal
ESCA	hsa-mir-125b-1&mature,MIMAT0000423	2	normal
ESCA	hsa-mir-125b-2&mature,MIMAT0000423	2	normal
ESCA	hsa-mir-99a&mature,MIMAT0000097	2	normal
ESCA	hsa-mir-99b&mature,MIMAT0000689	3	normal
HNSC	hsa-let-7a-1&mature,MIMAT0000062	7	tumor
HNSC	hsa-let-7a-2&mature,MIMAT0000062	6	tumor
HNSC	hsa-let-7a-2&mature,MIMAT0010195	1	tumor
HNSC	hsa-let-7a-3&mature,MIMAT0000062	9	tumor
HNSC	hsa-let-7b&mature,MIMAT0000063	7	tumor
HNSC	hsa-let-7b&mature,MIMAT0004482	2	tumor
HNSC	hsa-let-7c&mature,MIMAT0000064	9	tumor
HNSC	hsa-let-7c&mature,MIMAT0026472	2	tumor

HNSC	hsa-let-7d&mature,MIMAT0000065	4	tumor
HNSC	hsa-let-7d&mature,MIMAT0004484	4	tumor
HNSC	hsa-let-7e&mature,MIMAT0000066	5	tumor
HNSC	hsa-let-7f-1&mature,MIMAT0000067	7	tumor
HNSC	hsa-let-7f-2&mature,MIMAT0000067	7	tumor
HNSC	hsa-let-7g&mature,MIMAT0000414	5	tumor
HNSC	hsa-let-7g&mature,MIMAT0004584	1	tumor
HNSC	hsa-let-7i&mature,MIMAT0000415	4	tumor
HNSC	hsa-let-7i&mature,MIMAT0004585	3	tumor
HNSC	hsa-mir-100&mature,MIMAT0000098	9	tumor
HNSC	hsa-mir-10a&mature,MIMAT0000253	18	tumor
HNSC	hsa-mir-10b&mature,MIMAT0000254	15	tumor
HNSC	hsa-mir-10b&mature,MIMAT0004556	1	tumor
HNSC	hsa-mir-125a&mature,MIMAT0000443	4	tumor
HNSC	hsa-mir-125b-1&mature,MIMAT0000423	3	tumor
HNSC	hsa-mir-125b-2&mature,MIMAT0000423	3	tumor
HNSC	hsa-mir-125b-2&mature,MIMAT0004603	3	tumor
HNSC	hsa-mir-98&mature,MIMAT0000096	3	tumor
HNSC	hsa-mir-99a&mature,MIMAT0000097	9	tumor
HNSC	hsa-mir-99b&mature,MIMAT0000689	8	tumor
HNSC	hsa-mir-99b&mature,MIMAT0004678	3	tumor
HNSC	hsa-let-7a-1&mature,MIMAT0000062	5	normal
HNSC	hsa-let-7a-2&mature,MIMAT0000062	5	normal
HNSC	hsa-let-7a-3&mature,MIMAT0000062	5	normal
HNSC	hsa-let-7b&mature,MIMAT0000063	7	normal
HNSC	hsa-let-7c&mature,MIMAT0000064	6	normal
HNSC	hsa-let-7c&mature,MIMAT0026472	1	normal
HNSC	hsa-let-7d&mature,MIMAT0000065	3	normal
HNSC	hsa-let-7d&mature,MIMAT0004484	2	normal
HNSC	hsa-let-7e&mature,MIMAT0000066	3	normal
HNSC	hsa-let-7f-1&mature,MIMAT0000067	5	normal
HNSC	hsa-let-7f-2&mature,MIMAT0000067	6	normal
HNSC	hsa-let-7g&mature,MIMAT0000414	4	normal
HNSC	hsa-let-7g&mature,MIMAT0004584	1	normal
HNSC	hsa-let-7i&mature,MIMAT0000415	3	normal
HNSC	hsa-let-7i&mature,MIMAT0004585	3	normal
HNSC	hsa-mir-100&mature,MIMAT0000098	6	normal
HNSC	hsa-mir-10a&mature,MIMAT0000253	14	normal
HNSC	hsa-mir-10b&mature,MIMAT0000254	15	normal
HNSC	hsa-mir-125a&mature,MIMAT0000443	4	normal
HNSC	hsa-mir-125b-1&mature,MIMAT0000423	3	normal
HNSC	hsa-mir-125b-2&mature,MIMAT0000423	3	normal
HNSC	hsa-mir-99a&mature,MIMAT0000097	4	normal
HNSC	hsa-mir-99b&mature,MIMAT0000689	6	normal

KICHhsa-let-7a-1&mature,MIMAT0000062	5	tumor
KICHhsa-let-7a-2&mature,MIMAT0000062	5	tumor
KICHhsa-let-7a-3&mature,MIMAT0000062	5	tumor
KICHhsa-let-7b&mature,MIMAT0000063	6	tumor
KICHhsa-let-7c&mature,MIMAT0000064	5	tumor
KICHhsa-let-7d&mature,MIMAT0000065	3	tumor
KICHhsa-let-7d&mature,MIMAT0004484	3	tumor
KICHhsa-let-7e&mature,MIMAT0000066	3	tumor
KICHhsa-let-7f-1&mature,MIMAT0000067	5	tumor
KICHhsa-let-7f-2&mature,MIMAT0000067	6	tumor
KICHhsa-let-7g&mature,MIMAT0000414	3	tumor
KICHhsa-let-7g&mature,MIMAT0004584	1	tumor
KICHhsa-let-7i&mature,MIMAT0000415	3	tumor
KICHhsa-let-7i&mature,MIMAT0004585	2	tumor
KICHhsa-mir-100&mature,MIMAT0000098	5	tumor
KICHhsa-mir-10a&mature,MIMAT0000253	21	tumor
KICHhsa-mir-10b&mature,MIMAT0000254	18	tumor
KICHhsa-mir-10b&mature,MIMAT0004556	2	tumor
KICHhsa-mir-125a&mature,MIMAT0000443	4	tumor
KICHhsa-mir-125b-1&mature,MIMAT0000423	4	tumor
KICHhsa-mir-125b-2&mature,MIMAT0000423	4	tumor
KICHhsa-mir-98&mature,MIMAT0000096	1	tumor
KICHhsa-mir-99a&mature,MIMAT0000097	3	tumor
KICHhsa-mir-99b&mature,MIMAT0000689	6	tumor
KICHhsa-mir-99b&mature,MIMAT0004678	1	tumor
KICHhsa-let-7a-1&mature,MIMAT0000062	5	normal
KICHhsa-let-7a-2&mature,MIMAT0000062	5	normal
KICHhsa-let-7a-3&mature,MIMAT0000062	5	normal
KICHhsa-let-7b&mature,MIMAT0000063	5	normal
KICHhsa-let-7c&mature,MIMAT0000064	5	normal
KICHhsa-let-7d&mature,MIMAT0000065	2	normal
KICHhsa-let-7d&mature,MIMAT0004484	2	normal
KICHhsa-let-7e&mature,MIMAT0000066	3	normal
KICHhsa-let-7f-1&mature,MIMAT0000067	3	normal
KICHhsa-let-7f-2&mature,MIMAT0000067	4	normal
KICHhsa-let-7g&mature,MIMAT0000414	2	normal
KICHhsa-let-7g&mature,MIMAT0004584	1	normal
KICHhsa-let-7i&mature,MIMAT0000415	3	normal
KICHhsa-mir-100&mature,MIMAT0000098	5	normal
KICHhsa-mir-10a&mature,MIMAT0000253	18	normal
KICHhsa-mir-10b&mature,MIMAT0000254	17	normal
KICHhsa-mir-10b&mature,MIMAT0004556	2	normal
KICHhsa-mir-125a&mature,MIMAT0000443	4	normal
KICHhsa-mir-125b-1&mature,MIMAT0000423	2	normal

KICH	hsa-mir-125b-2&mature,MIMAT0000423	3	normal
KICH	hsa-mir-98&mature,MIMAT0000096	1	normal
KICH	hsa-mir-99a&mature,MIMAT0000097	3	normal
KICH	hsa-mir-99b&mature,MIMAT0000689	6	normal
KICH	hsa-mir-99b&mature,MIMAT0004678	1	normal
KIRC	hsa-let-7a-1&mature,MIMAT0000062	6	tumor
KIRC	hsa-let-7a-2&mature,MIMAT0000062	6	tumor
KIRC	hsa-let-7a-2&mature,MIMAT0010195	2	tumor
KIRC	hsa-let-7a-3&mature,MIMAT0000062	8	tumor
KIRC	hsa-let-7b&mature,MIMAT0000063	9	tumor
KIRC	hsa-let-7b&mature,MIMAT0004482	1	tumor
KIRC	hsa-let-7c&mature,MIMAT0000064	5	tumor
KIRC	hsa-let-7c&mature,MIMAT0026472	1	tumor
KIRC	hsa-let-7d&mature,MIMAT0000065	5	tumor
KIRC	hsa-let-7d&mature,MIMAT0004484	5	tumor
KIRC	hsa-let-7e&mature,MIMAT0000066	4	tumor
KIRC	hsa-let-7e&mature,MIMAT0004485	1	tumor
KIRC	hsa-let-7f-1&mature,MIMAT0000067	8	tumor
KIRC	hsa-let-7f-2&mature,MIMAT0000067	7	tumor
KIRC	hsa-let-7g&mature,MIMAT0000414	5	tumor
KIRC	hsa-let-7g&mature,MIMAT0004584	1	tumor
KIRC	hsa-let-7i&mature,MIMAT0000415	3	tumor
KIRC	hsa-let-7i&mature,MIMAT0004585	3	tumor
KIRC	hsa-mir-100&mature,MIMAT0000098	13	tumor
KIRC	hsa-mir-10a&mature,MIMAT0000253	25	tumor
KIRC	hsa-mir-10b&mature,MIMAT0000254	25	tumor
KIRC	hsa-mir-10b&mature,MIMAT0004556	1	tumor
KIRC	hsa-mir-125a&mature,MIMAT0000443	5	tumor
KIRC	hsa-mir-125b-1&mature,MIMAT0000423	3	tumor
KIRC	hsa-mir-125b-2&mature,MIMAT0000423	3	tumor
KIRC	hsa-mir-98&mature,MIMAT0000096	3	tumor
KIRC	hsa-mir-99a&mature,MIMAT0000097	7	tumor
KIRC	hsa-mir-99b&mature,MIMAT0000689	10	tumor
KIRC	hsa-mir-99b&mature,MIMAT0004678	4	tumor
KIRC	hsa-let-7a-1&mature,MIMAT0000062	5	normal
KIRC	hsa-let-7a-2&mature,MIMAT0000062	5	normal
KIRC	hsa-let-7a-3&mature,MIMAT0000062	5	normal
KIRC	hsa-let-7b&mature,MIMAT0000063	5	normal
KIRC	hsa-let-7c&mature,MIMAT0000064	5	normal
KIRC	hsa-let-7c&mature,MIMAT0026472	1	normal
KIRC	hsa-let-7d&mature,MIMAT0000065	2	normal
KIRC	hsa-let-7d&mature,MIMAT0004484	2	normal
KIRC	hsa-let-7e&mature,MIMAT0000066	4	normal
KIRC	hsa-let-7f-1&mature,MIMAT0000067	4	normal

KIRC	hsa-let-7f-2&mature,MIMAT0000067	4	normal
KIRC	hsa-let-7g&mature,MIMAT0000414	2	normal
KIRC	hsa-let-7g&mature,MIMAT0004584	1	normal
KIRC	hsa-let-7i&mature,MIMAT0000415	3	normal
KIRC	hsa-let-7i&mature,MIMAT0004585	2	normal
KIRC	hsa-mir-100&mature,MIMAT0000098	4	normal
KIRC	hsa-mir-10a&mature,MIMAT0000253	17	normal
KIRC	hsa-mir-10b&mature,MIMAT0000254	22	normal
KIRC	hsa-mir-10b&mature,MIMAT0004556	1	normal
KIRC	hsa-mir-125a&mature,MIMAT0000443	4	normal
KIRC	hsa-mir-125b-1&mature,MIMAT0000423	3	normal
KIRC	hsa-mir-125b-2&mature,MIMAT0000423	3	normal
KIRC	hsa-mir-98&mature,MIMAT0000096	1	normal
KIRC	hsa-mir-99a&mature,MIMAT0000097	3	normal
KIRC	hsa-mir-99b&mature,MIMAT0000689	9	normal
KIRP	hsa-let-7a-1&mature,MIMAT0000062	6	tumor
KIRP	hsa-let-7a-2&mature,MIMAT0000062	6	tumor
KIRP	hsa-let-7a-3&mature,MIMAT0000062	8	tumor
KIRP	hsa-let-7b&mature,MIMAT0000063	5	tumor
KIRP	hsa-let-7b&mature,MIMAT0004482	1	tumor
KIRP	hsa-let-7c&mature,MIMAT0000064	5	tumor
KIRP	hsa-let-7c&mature,MIMAT0026472	1	tumor
KIRP	hsa-let-7d&mature,MIMAT0000065	3	tumor
KIRP	hsa-let-7d&mature,MIMAT0004484	3	tumor
KIRP	hsa-let-7e&mature,MIMAT0000066	4	tumor
KIRP	hsa-let-7f-1&mature,MIMAT0000067	7	tumor
KIRP	hsa-let-7f-2&mature,MIMAT0000067	6	tumor
KIRP	hsa-let-7g&mature,MIMAT0000414	3	tumor
KIRP	hsa-let-7g&mature,MIMAT0004584	1	tumor
KIRP	hsa-let-7i&mature,MIMAT0000415	3	tumor
KIRP	hsa-let-7i&mature,MIMAT0004585	3	tumor
KIRP	hsa-mir-100&mature,MIMAT0000098	7	tumor
KIRP	hsa-mir-10a&mature,MIMAT0000253	19	tumor
KIRP	hsa-mir-10b&mature,MIMAT0000254	19	tumor
KIRP	hsa-mir-10b&mature,MIMAT0004556	2	tumor
KIRP	hsa-mir-125a&mature,MIMAT0000443	4	tumor
KIRP	hsa-mir-125b-1&mature,MIMAT0000423	3	tumor
KIRP	hsa-mir-125b-2&mature,MIMAT0000423	3	tumor
KIRP	hsa-mir-98&mature,MIMAT0000096	2	tumor
KIRP	hsa-mir-99a&mature,MIMAT0000097	3	tumor
KIRP	hsa-mir-99b&mature,MIMAT0000689	8	tumor
KIRP	hsa-mir-99b&mature,MIMAT0004678	2	tumor
KIRP	hsa-let-7a-1&mature,MIMAT0000062	5	normal
KIRP	hsa-let-7a-2&mature,MIMAT0000062	5	normal

KIRP	hsa-let-7a-3&mature,MIMAT0000062	5	normal
KIRP	hsa-let-7b&mature,MIMAT0000063	5	normal
KIRP	hsa-let-7c&mature,MIMAT0000064	5	normal
KIRP	hsa-let-7d&mature,MIMAT0000065	2	normal
KIRP	hsa-let-7d&mature,MIMAT0004484	2	normal
KIRP	hsa-let-7e&mature,MIMAT0000066	4	normal
KIRP	hsa-let-7f-1&mature,MIMAT0000067	4	normal
KIRP	hsa-let-7f-2&mature,MIMAT0000067	5	normal
KIRP	hsa-let-7g&mature,MIMAT0000414	3	normal
KIRP	hsa-let-7g&mature,MIMAT0004584	1	normal
KIRP	hsa-let-7i&mature,MIMAT0000415	3	normal
KIRP	hsa-let-7i&mature,MIMAT0004585	2	normal
KIRP	hsa-mir-100&mature,MIMAT0000098	5	normal
KIRP	hsa-mir-10a&mature,MIMAT0000253	18	normal
KIRP	hsa-mir-10b&mature,MIMAT0000254	20	normal
KIRP	hsa-mir-10b&mature,MIMAT0004556	1	normal
KIRP	hsa-mir-125a&mature,MIMAT0000443	4	normal
KIRP	hsa-mir-125b-1&mature,MIMAT0000423	2	normal
KIRP	hsa-mir-125b-2&mature,MIMAT0000423	2	normal
KIRP	hsa-mir-99a&mature,MIMAT0000097	3	normal
KIRP	hsa-mir-99b&mature,MIMAT0000689	6	normal
LIHC	hsa-let-7a-1&mature,MIMAT0000062	6	tumor
LIHC	hsa-let-7a-2&mature,MIMAT0000062	5	tumor
LIHC	hsa-let-7a-3&mature,MIMAT0000062	7	tumor
LIHC	hsa-let-7b&mature,MIMAT0000063	6	tumor
LIHC	hsa-let-7b&mature,MIMAT0004482	1	tumor
LIHC	hsa-let-7c&mature,MIMAT0000064	7	tumor
LIHC	hsa-let-7c&mature,MIMAT0026472	1	tumor
LIHC	hsa-let-7d&mature,MIMAT0000065	4	tumor
LIHC	hsa-let-7d&mature,MIMAT0004484	4	tumor
LIHC	hsa-let-7e&mature,MIMAT0000066	5	tumor
LIHC	hsa-let-7f-1&mature,MIMAT0000067	7	tumor
LIHC	hsa-let-7f-2&mature,MIMAT0000067	8	tumor
LIHC	hsa-let-7g&mature,MIMAT0000414	5	tumor
LIHC	hsa-let-7g&mature,MIMAT0004584	1	tumor
LIHC	hsa-let-7i&mature,MIMAT0000415	3	tumor
LIHC	hsa-let-7i&mature,MIMAT0004585	2	tumor
LIHC	hsa-mir-100&mature,MIMAT0000098	7	tumor
LIHC	hsa-mir-10a&mature,MIMAT0000253	14	tumor
LIHC	hsa-mir-10b&mature,MIMAT0000254	13	tumor
LIHC	hsa-mir-125a&mature,MIMAT0000443	4	tumor
LIHC	hsa-mir-125b-1&mature,MIMAT0000423	3	tumor
LIHC	hsa-mir-125b-2&mature,MIMAT0000423	3	tumor
LIHC	hsa-mir-125b-2&mature,MIMAT0004603	1	tumor

LIHC	hsa-mir-98&mature,MIMAT0000096	3	tumor
LIHC	hsa-mir-99a&mature,MIMAT0000097	4	tumor
LIHC	hsa-mir-99b&mature,MIMAT0000689	7	tumor
LIHC	hsa-mir-99b&mature,MIMAT0004678	4	tumor
LIHC	hsa-let-7a-1&mature,MIMAT0000062	5	normal
LIHC	hsa-let-7a-2&mature,MIMAT0000062	5	normal
LIHC	hsa-let-7a-3&mature,MIMAT0000062	5	normal
LIHC	hsa-let-7b&mature,MIMAT0000063	6	normal
LIHC	hsa-let-7c&mature,MIMAT0000064	6	normal
LIHC	hsa-let-7d&mature,MIMAT0000065	3	normal
LIHC	hsa-let-7d&mature,MIMAT0004484	3	normal
LIHC	hsa-let-7e&mature,MIMAT0000066	3	normal
LIHC	hsa-let-7f-1&mature,MIMAT0000067	6	normal
LIHC	hsa-let-7f-2&mature,MIMAT0000067	6	normal
LIHC	hsa-let-7g&mature,MIMAT0000414	4	normal
LIHC	hsa-let-7g&mature,MIMAT0004584	1	normal
LIHC	hsa-let-7i&mature,MIMAT0000415	3	normal
LIHC	hsa-let-7i&mature,MIMAT0004585	1	normal
LIHC	hsa-mir-100&mature,MIMAT0000098	6	normal
LIHC	hsa-mir-10a&mature,MIMAT0000253	18	normal
LIHC	hsa-mir-10b&mature,MIMAT0000254	8	normal
LIHC	hsa-mir-125a&mature,MIMAT0000443	4	normal
LIHC	hsa-mir-125b-1&mature,MIMAT0000423	3	normal
LIHC	hsa-mir-125b-2&mature,MIMAT0000423	3	normal
LIHC	hsa-mir-99a&mature,MIMAT0000097	3	normal
LIHC	hsa-mir-99b&mature,MIMAT0000689	5	normal
LUAD	hsa-let-7a-1&mature,MIMAT0000062	5	tumor
LUAD	hsa-let-7a-2&mature,MIMAT0000062	5	tumor
LUAD	hsa-let-7a-2&mature,MIMAT0010195	2	tumor
LUAD	hsa-let-7a-3&mature,MIMAT0000062	7	tumor
LUAD	hsa-let-7b&mature,MIMAT0000063	9	tumor
LUAD	hsa-let-7b&mature,MIMAT0004482	1	tumor
LUAD	hsa-let-7c&mature,MIMAT0000064	5	tumor
LUAD	hsa-let-7c&mature,MIMAT0026472	1	tumor
LUAD	hsa-let-7d&mature,MIMAT0000065	4	tumor
LUAD	hsa-let-7d&mature,MIMAT0004484	4	tumor
LUAD	hsa-let-7e&mature,MIMAT0000066	6	tumor
LUAD	hsa-let-7e&mature,MIMAT0004485	1	tumor
LUAD	hsa-let-7f-1&mature,MIMAT0000067	7	tumor
LUAD	hsa-let-7f-2&mature,MIMAT0000067	7	tumor
LUAD	hsa-let-7g&mature,MIMAT0000414	5	tumor
LUAD	hsa-let-7g&mature,MIMAT0004584	1	tumor
LUAD	hsa-let-7i&mature,MIMAT0000415	6	tumor
LUAD	hsa-let-7i&mature,MIMAT0004585	3	tumor

LUAD	hsa-mir-100&mature,MIMAT0000098	15	tumor
LUAD	hsa-mir-10a&mature,MIMAT0000253	30	tumor
LUAD	hsa-mir-10b&mature,MIMAT0000254	20	tumor
LUAD	hsa-mir-10b&mature,MIMAT0004556	1	tumor
LUAD	hsa-mir-125a&mature,MIMAT0000443	4	tumor
LUAD	hsa-mir-125b-1&mature,MIMAT0000423	3	tumor
LUAD	hsa-mir-125b-2&mature,MIMAT0000423	3	tumor
LUAD	hsa-mir-98&mature,MIMAT0000096	3	tumor
LUAD	hsa-mir-99a&mature,MIMAT0000097	3	tumor
LUAD	hsa-mir-99b&mature,MIMAT0000689	12	tumor
LUAD	hsa-mir-99b&mature,MIMAT0004678	4	tumor
LUAD	hsa-let-7a-1&mature,MIMAT0000062	6	normal
LUAD	hsa-let-7a-2&mature,MIMAT0000062	6	normal
LUAD	hsa-let-7a-3&mature,MIMAT0000062	9	normal
LUAD	hsa-let-7b&mature,MIMAT0000063	11	normal
LUAD	hsa-let-7b&mature,MIMAT0004482	2	normal
LUAD	hsa-let-7c&mature,MIMAT0000064	6	normal
LUAD	hsa-let-7d&mature,MIMAT0000065	3	normal
LUAD	hsa-let-7d&mature,MIMAT0004484	8	normal
LUAD	hsa-let-7e&mature,MIMAT0000066	5	normal
LUAD	hsa-let-7e&mature,MIMAT0004485	1	normal
LUAD	hsa-let-7f-1&mature,MIMAT0000067	6	normal
LUAD	hsa-let-7f-2&mature,MIMAT0000067	6	normal
LUAD	hsa-let-7g&mature,MIMAT0000414	4	normal
LUAD	hsa-let-7i&mature,MIMAT0000415	3	normal
LUAD	hsa-let-7i&mature,MIMAT0004585	3	normal
LUAD	hsa-mir-100&mature,MIMAT0000098	6	normal
LUAD	hsa-mir-10a&mature,MIMAT0000253	17	normal
LUAD	hsa-mir-10b&mature,MIMAT0000254	14	normal
LUAD	hsa-mir-125a&mature,MIMAT0000443	5	normal
LUAD	hsa-mir-125b-1&mature,MIMAT0000423	4	normal
LUAD	hsa-mir-125b-2&mature,MIMAT0000423	3	normal
LUAD	hsa-mir-98&mature,MIMAT0000096	1	normal
LUAD	hsa-mir-99a&mature,MIMAT0000097	3	normal
LUAD	hsa-mir-99b&mature,MIMAT0000689	10	normal
LUAD	hsa-mir-99b&mature,MIMAT0004678	3	normal
LUSC	hsa-let-7a-1&mature,MIMAT0000062	6	tumor
LUSC	hsa-let-7a-2&mature,MIMAT0000062	7	tumor
LUSC	hsa-let-7a-2&mature,MIMAT0010195	1	tumor
LUSC	hsa-let-7a-3&mature,MIMAT0000062	8	tumor
LUSC	hsa-let-7b&mature,MIMAT0000063	10	tumor
LUSC	hsa-let-7b&mature,MIMAT0004482	1	tumor
LUSC	hsa-let-7c&mature,MIMAT0000064	9	tumor
LUSC	hsa-let-7c&mature,MIMAT0026472	1	tumor

LUSC	hsa-let-7d&mature,MIMAT0000065	4	tumor
LUSC	hsa-let-7d&mature,MIMAT0004484	4	tumor
LUSC	hsa-let-7e&mature,MIMAT0000066	4	tumor
LUSC	hsa-let-7e&mature,MIMAT0004485	1	tumor
LUSC	hsa-let-7f-1&mature,MIMAT0000067	7	tumor
LUSC	hsa-let-7f-2&mature,MIMAT0000067	6	tumor
LUSC	hsa-let-7g&mature,MIMAT0000414	4	tumor
LUSC	hsa-let-7g&mature,MIMAT0004584	1	tumor
LUSC	hsa-let-7i&mature,MIMAT0000415	3	tumor
LUSC	hsa-let-7i&mature,MIMAT0004585	3	tumor
LUSC	hsa-mir-100&mature,MIMAT0000098	12	tumor
LUSC	hsa-mir-10a&mature,MIMAT0000253	28	tumor
LUSC	hsa-mir-10b&mature,MIMAT0000254	19	tumor
LUSC	hsa-mir-10b&mature,MIMAT0004556	1	tumor
LUSC	hsa-mir-125a&mature,MIMAT0000443	4	tumor
LUSC	hsa-mir-125b-1&mature,MIMAT0000423	3	tumor
LUSC	hsa-mir-125b-2&mature,MIMAT0000423	3	tumor
LUSC	hsa-mir-125b-2&mature,MIMAT0004603	4	tumor
LUSC	hsa-mir-98&mature,MIMAT0000096	3	tumor
LUSC	hsa-mir-99a&mature,MIMAT0000097	9	tumor
LUSC	hsa-mir-99b&mature,MIMAT0000689	10	tumor
LUSC	hsa-mir-99b&mature,MIMAT0004678	5	tumor
LUSC	hsa-let-7a-1&mature,MIMAT0000062	5	normal
LUSC	hsa-let-7a-2&mature,MIMAT0000062	5	normal
LUSC	hsa-let-7a-3&mature,MIMAT0000062	5	normal
LUSC	hsa-let-7b&mature,MIMAT0000063	6	normal
LUSC	hsa-let-7b&mature,MIMAT0004482	1	normal
LUSC	hsa-let-7c&mature,MIMAT0000064	5	normal
LUSC	hsa-let-7d&mature,MIMAT0000065	3	normal
LUSC	hsa-let-7d&mature,MIMAT0004484	3	normal
LUSC	hsa-let-7e&mature,MIMAT0000066	3	normal
LUSC	hsa-let-7f-1&mature,MIMAT0000067	4	normal
LUSC	hsa-let-7f-2&mature,MIMAT0000067	5	normal
LUSC	hsa-let-7g&mature,MIMAT0000414	2	normal
LUSC	hsa-let-7g&mature,MIMAT0004584	1	normal
LUSC	hsa-let-7i&mature,MIMAT0000415	3	normal
LUSC	hsa-let-7i&mature,MIMAT0004585	3	normal
LUSC	hsa-mir-100&mature,MIMAT0000098	6	normal
LUSC	hsa-mir-10a&mature,MIMAT0000253	16	normal
LUSC	hsa-mir-10b&mature,MIMAT0000254	10	normal
LUSC	hsa-mir-125a&mature,MIMAT0000443	4	normal
LUSC	hsa-mir-125b-1&mature,MIMAT0000423	2	normal
LUSC	hsa-mir-125b-2&mature,MIMAT0000423	2	normal
LUSC	hsa-mir-99a&mature,MIMAT0000097	3	normal

LUSC	hsa-mir-99b&mature,MIMAT0000689	6	normal
LUSC	hsa-mir-99b&mature,MIMAT0004678	1	normal
PRAD	hsa-let-7a-1&mature,MIMAT0000062	6	tumor
PRAD	hsa-let-7a-2&mature,MIMAT0000062	5	tumor
PRAD	hsa-let-7a-3&mature,MIMAT0000062	7	tumor
PRAD	hsa-let-7b&mature,MIMAT0000063	6	tumor
PRAD	hsa-let-7b&mature,MIMAT0004482	1	tumor
PRAD	hsa-let-7c&mature,MIMAT0000064	8	tumor
PRAD	hsa-let-7c&mature,MIMAT0026472	2	tumor
PRAD	hsa-let-7d&mature,MIMAT0000065	3	tumor
PRAD	hsa-let-7d&mature,MIMAT0004484	3	tumor
PRAD	hsa-let-7e&mature,MIMAT0000066	4	tumor
PRAD	hsa-let-7f-1&mature,MIMAT0000067	5	tumor
PRAD	hsa-let-7f-2&mature,MIMAT0000067	5	tumor
PRAD	hsa-let-7g&mature,MIMAT0000414	4	tumor
PRAD	hsa-let-7g&mature,MIMAT0004584	1	tumor
PRAD	hsa-let-7i&mature,MIMAT0000415	3	tumor
PRAD	hsa-let-7i&mature,MIMAT0004585	1	tumor
PRAD	hsa-mir-100&mature,MIMAT0000098	7	tumor
PRAD	hsa-mir-10a&mature,MIMAT0000253	11	tumor
PRAD	hsa-mir-10b&mature,MIMAT0000254	13	tumor
PRAD	hsa-mir-10b&mature,MIMAT0004556	1	tumor
PRAD	hsa-mir-125a&mature,MIMAT0000443	4	tumor
PRAD	hsa-mir-125b-1&mature,MIMAT0000423	5	tumor
PRAD	hsa-mir-125b-2&mature,MIMAT0000423	4	tumor
PRAD	hsa-mir-125b-2&mature,MIMAT0004603	1	tumor
PRAD	hsa-mir-98&mature,MIMAT0000096	2	tumor
PRAD	hsa-mir-99a&mature,MIMAT0000097	7	tumor
PRAD	hsa-mir-99b&mature,MIMAT0000689	5	tumor
PRAD	hsa-mir-99b&mature,MIMAT0004678	1	tumor
PRAD	hsa-let-7a-1&mature,MIMAT0000062	5	normal
PRAD	hsa-let-7a-2&mature,MIMAT0000062	5	normal
PRAD	hsa-let-7a-3&mature,MIMAT0000062	5	normal
PRAD	hsa-let-7b&mature,MIMAT0000063	6	normal
PRAD	hsa-let-7c&mature,MIMAT0000064	6	normal
PRAD	hsa-let-7d&mature,MIMAT0000065	1	normal
PRAD	hsa-let-7d&mature,MIMAT0004484	3	normal
PRAD	hsa-let-7e&mature,MIMAT0000066	3	normal
PRAD	hsa-let-7f-1&mature,MIMAT0000067	3	normal
PRAD	hsa-let-7f-2&mature,MIMAT0000067	4	normal
PRAD	hsa-let-7g&mature,MIMAT0000414	2	normal
PRAD	hsa-let-7g&mature,MIMAT0004584	1	normal
PRAD	hsa-let-7i&mature,MIMAT0000415	2	normal
PRAD	hsa-mir-100&mature,MIMAT0000098	6	normal

PRAD	hsa-mir-10a&mature,MIMAT0000253	19	normal
PRAD	hsa-mir-10b&mature,MIMAT0000254	16	normal
PRAD	hsa-mir-125a&mature,MIMAT0000443	3	normal
PRAD	hsa-mir-125b-1&mature,MIMAT0000423	3	normal
PRAD	hsa-mir-125b-2&mature,MIMAT0000423	3	normal
PRAD	hsa-mir-99a&mature,MIMAT0000097	4	normal
PRAD	hsa-mir-99b&mature,MIMAT0000689	5	normal
STAD	hsa-let-7a-1&mature,MIMAT0000062	5	tumor
STAD	hsa-let-7a-2&mature,MIMAT0000062	5	tumor
STAD	hsa-let-7a-3&mature,MIMAT0000062	6	tumor
STAD	hsa-let-7b&mature,MIMAT0000063	6	tumor
STAD	hsa-let-7b&mature,MIMAT0004482	1	tumor
STAD	hsa-let-7c&mature,MIMAT0000064	5	tumor
STAD	hsa-let-7c&mature,MIMAT0026472	1	tumor
STAD	hsa-let-7d&mature,MIMAT0000065	3	tumor
STAD	hsa-let-7d&mature,MIMAT0004484	4	tumor
STAD	hsa-let-7e&mature,MIMAT0000066	5	tumor
STAD	hsa-let-7f-1&mature,MIMAT0000067	5	tumor
STAD	hsa-let-7f-2&mature,MIMAT0000067	5	tumor
STAD	hsa-let-7g&mature,MIMAT0000414	4	tumor
STAD	hsa-let-7g&mature,MIMAT0004584	1	tumor
STAD	hsa-let-7i&mature,MIMAT0000415	3	tumor
STAD	hsa-let-7i&mature,MIMAT0004585	3	tumor
STAD	hsa-mir-100&mature,MIMAT0000098	7	tumor
STAD	hsa-mir-10a&mature,MIMAT0000253	25	tumor
STAD	hsa-mir-10b&mature,MIMAT0000254	14	tumor
STAD	hsa-mir-125a&mature,MIMAT0000443	4	tumor
STAD	hsa-mir-125b-1&mature,MIMAT0000423	3	tumor
STAD	hsa-mir-125b-2&mature,MIMAT0000423	3	tumor
STAD	hsa-mir-125b-2&mature,MIMAT0004603	1	tumor
STAD	hsa-mir-98&mature,MIMAT0000096	3	tumor
STAD	hsa-mir-99a&mature,MIMAT0000097	3	tumor
STAD	hsa-mir-99b&mature,MIMAT0000689	11	tumor
STAD	hsa-mir-99b&mature,MIMAT0004678	4	tumor
STAD	hsa-let-7a-1&mature,MIMAT0000062	5	normal
STAD	hsa-let-7a-2&mature,MIMAT0000062	5	normal
STAD	hsa-let-7a-3&mature,MIMAT0000062	5	normal
STAD	hsa-let-7b&mature,MIMAT0000063	5	normal
STAD	hsa-let-7c&mature,MIMAT0000064	5	normal
STAD	hsa-let-7d&mature,MIMAT0000065	3	normal
STAD	hsa-let-7d&mature,MIMAT0004484	3	normal
STAD	hsa-let-7e&mature,MIMAT0000066	4	normal
STAD	hsa-let-7f-1&mature,MIMAT0000067	6	normal
STAD	hsa-let-7f-2&mature,MIMAT0000067	6	normal

STAD	hsa-let-7g&mature,MIMAT0000414	3	normal
STAD	hsa-let-7g&mature,MIMAT0004584	1	normal
STAD	hsa-let-7i&mature,MIMAT0000415	3	normal
STAD	hsa-let-7i&mature,MIMAT0004585	3	normal
STAD	hsa-mir-100&mature,MIMAT0000098	6	normal
STAD	hsa-mir-10a&mature,MIMAT0000253	17	normal
STAD	hsa-mir-10b&mature,MIMAT0000254	10	normal
STAD	hsa-mir-125a&mature,MIMAT0000443	4	normal
STAD	hsa-mir-125b-1&mature,MIMAT0000423	3	normal
STAD	hsa-mir-125b-2&mature,MIMAT0000423	3	normal
STAD	hsa-mir-99a&mature,MIMAT0000097	3	normal
STAD	hsa-mir-99b&mature,MIMAT0000689	6	normal
THCA	hsa-let-7a-1&mature,MIMAT0000062	6	tumor
THCA	hsa-let-7a-2&mature,MIMAT0000062	6	tumor
THCA	hsa-let-7a-3&mature,MIMAT0000062	7	tumor
THCA	hsa-let-7b&mature,MIMAT0000063	6	tumor
THCA	hsa-let-7b&mature,MIMAT0004482	1	tumor
THCA	hsa-let-7c&mature,MIMAT0000064	9	tumor
THCA	hsa-let-7c&mature,MIMAT0026472	2	tumor
THCA	hsa-let-7d&mature,MIMAT0000065	3	tumor
THCA	hsa-let-7d&mature,MIMAT0004484	5	tumor
THCA	hsa-let-7e&mature,MIMAT0000066	6	tumor
THCA	hsa-let-7e&mature,MIMAT0004485	1	tumor
THCA	hsa-let-7f-1&mature,MIMAT0000067	6	tumor
THCA	hsa-let-7f-2&mature,MIMAT0000067	6	tumor
THCA	hsa-let-7g&mature,MIMAT0000414	6	tumor
THCA	hsa-let-7g&mature,MIMAT0004584	1	tumor
THCA	hsa-let-7i&mature,MIMAT0000415	8	tumor
THCA	hsa-let-7i&mature,MIMAT0004585	4	tumor
THCA	hsa-mir-100&mature,MIMAT0000098	9	tumor
THCA	hsa-mir-10a&mature,MIMAT0000253	14	tumor
THCA	hsa-mir-10b&mature,MIMAT0000254	13	tumor
THCA	hsa-mir-125a&mature,MIMAT0000443	5	tumor
THCA	hsa-mir-125b-1&mature,MIMAT0000423	5	tumor
THCA	hsa-mir-125b-2&mature,MIMAT0000423	5	tumor
THCA	hsa-mir-125b-2&mature,MIMAT0004603	4	tumor
THCA	hsa-mir-98&mature,MIMAT0000096	3	tumor
THCA	hsa-mir-99a&mature,MIMAT0000097	8	tumor
THCA	hsa-mir-99b&mature,MIMAT0000689	10	tumor
THCA	hsa-mir-99b&mature,MIMAT0004678	5	tumor
THCA	hsa-let-7a-1&mature,MIMAT0000062	5	normal
THCA	hsa-let-7a-2&mature,MIMAT0000062	5	normal
THCA	hsa-let-7a-3&mature,MIMAT0000062	5	normal
THCA	hsa-let-7b&mature,MIMAT0000063	6	normal

THCA	hsa-let-7b&mature,MIMAT0004482	1	normal
THCA	hsa-let-7c&mature,MIMAT0000064	7	normal
THCA	hsa-let-7c&mature,MIMAT0026472	1	normal
THCA	hsa-let-7d&mature,MIMAT0000065	4	normal
THCA	hsa-let-7d&mature,MIMAT0004484	5	normal
THCA	hsa-let-7e&mature,MIMAT0000066	6	normal
THCA	hsa-let-7f-1&mature,MIMAT0000067	6	normal
THCA	hsa-let-7f-2&mature,MIMAT0000067	6	normal
THCA	hsa-let-7g&mature,MIMAT0000414	5	normal
THCA	hsa-let-7g&mature,MIMAT0004584	1	normal
THCA	hsa-let-7i&mature,MIMAT0000415	6	normal
THCA	hsa-let-7i&mature,MIMAT0004585	3	normal
THCA	hsa-mir-100&mature,MIMAT0000098	10	normal
THCA	hsa-mir-10a&mature,MIMAT0000253	14	normal
THCA	hsa-mir-10b&mature,MIMAT0000254	14	normal
THCA	hsa-mir-125a&mature,MIMAT0000443	6	normal
THCA	hsa-mir-125b-1&mature,MIMAT0000423	5	normal
THCA	hsa-mir-125b-2&mature,MIMAT0000423	5	normal
THCA	hsa-mir-125b-2&mature,MIMAT0004603	1	normal
THCA	hsa-mir-98&mature,MIMAT0000096	1	normal
THCA	hsa-mir-99a&mature,MIMAT0000097	6	normal
THCA	hsa-mir-99b&mature,MIMAT0000689	10	normal
THCA	hsa-mir-99b&mature,MIMAT0004678	3	normal
UCEC	hsa-let-7a-1&mature,MIMAT0000062	8	tumor
UCEC	hsa-let-7a-1&mature,MIMAT0004481	2	tumor
UCEC	hsa-let-7a-2&mature,MIMAT0000062	7	tumor
UCEC	hsa-let-7a-2&mature,MIMAT0010195	2	tumor
UCEC	hsa-let-7a-3&mature,MIMAT0000062	10	tumor
UCEC	hsa-let-7a-3&mature,MIMAT0004481	2	tumor
UCEC	hsa-let-7b&mature,MIMAT0000063	13	tumor
UCEC	hsa-let-7b&mature,MIMAT0004482	2	tumor
UCEC	hsa-let-7c&mature,MIMAT0000064	9	tumor
UCEC	hsa-let-7c&mature,MIMAT0026472	4	tumor
UCEC	hsa-let-7d&mature,MIMAT0000065	5	tumor
UCEC	hsa-let-7d&mature,MIMAT0004484	4	tumor
UCEC	hsa-let-7e&mature,MIMAT0000066	4	tumor
UCEC	hsa-let-7e&mature,MIMAT0004485	1	tumor
UCEC	hsa-let-7f-1&mature,MIMAT0000067	5	tumor
UCEC	hsa-let-7f-2&mature,MIMAT0000067	6	tumor
UCEC	hsa-let-7g&mature,MIMAT0000414	7	tumor
UCEC	hsa-let-7g&mature,MIMAT0004584	1	tumor
UCEC	hsa-let-7i&mature,MIMAT0000415	4	tumor
UCEC	hsa-let-7i&mature,MIMAT0004585	3	tumor
UCEC	hsa-mir-100&mature,MIMAT0000098	12	tumor

UCEC	hsa-mir-10a&mature,MIMAT0000253	34	tumor
UCEC	hsa-mir-10a&mature,MIMAT0004555	1	tumor
UCEC	hsa-mir-10b&mature,MIMAT0000254	29	tumor
UCEC	hsa-mir-10b&mature,MIMAT0004556	3	tumor
UCEC	hsa-mir-125a&mature,MIMAT0000443	5	tumor
UCEC	hsa-mir-125b-1&mature,MIMAT0000423	5	tumor
UCEC	hsa-mir-125b-2&mature,MIMAT0000423	5	tumor
UCEC	hsa-mir-125b-2&mature,MIMAT0004603	4	tumor
UCEC	hsa-mir-98&mature,MIMAT0000096	3	tumor
UCEC	hsa-mir-99a&mature,MIMAT0000097	9	tumor
UCEC	hsa-mir-99a&mature,MIMAT0004511	2	tumor
UCEC	hsa-mir-99b&mature,MIMAT0000689	12	tumor
UCEC	hsa-mir-99b&mature,MIMAT0004678	7	tumor
UCEC	hsa-let-7a-1&mature,MIMAT0000062	5	normal
UCEC	hsa-let-7a-2&mature,MIMAT0000062	5	normal
UCEC	hsa-let-7a-3&mature,MIMAT0000062	5	normal
UCEC	hsa-let-7b&mature,MIMAT0000063	6	normal
UCEC	hsa-let-7b&mature,MIMAT0004482	1	normal
UCEC	hsa-let-7c&mature,MIMAT0000064	5	normal
UCEC	hsa-let-7c&mature,MIMAT0026472	1	normal
UCEC	hsa-let-7d&mature,MIMAT0000065	2	normal
UCEC	hsa-let-7d&mature,MIMAT0004484	2	normal
UCEC	hsa-let-7e&mature,MIMAT0000066	3	normal
UCEC	hsa-let-7f-1&mature,MIMAT0000067	3	normal
UCEC	hsa-let-7f-2&mature,MIMAT0000067	4	normal
UCEC	hsa-let-7g&mature,MIMAT0000414	3	normal
UCEC	hsa-let-7g&mature,MIMAT0004584	1	normal
UCEC	hsa-let-7i&mature,MIMAT0000415	3	normal
UCEC	hsa-let-7i&mature,MIMAT0004585	1	normal
UCEC	hsa-mir-100&mature,MIMAT0000098	6	normal
UCEC	hsa-mir-10a&mature,MIMAT0000253	18	normal
UCEC	hsa-mir-10b&mature,MIMAT0000254	22	normal
UCEC	hsa-mir-125a&mature,MIMAT0000443	5	normal
UCEC	hsa-mir-125b-1&mature,MIMAT0000423	4	normal
UCEC	hsa-mir-125b-2&mature,MIMAT0000423	4	normal
UCEC	hsa-mir-99a&mature,MIMAT0000097	6	normal
UCEC	hsa-mir-99b&mature,MIMAT0000689	8	normal

Figure S1

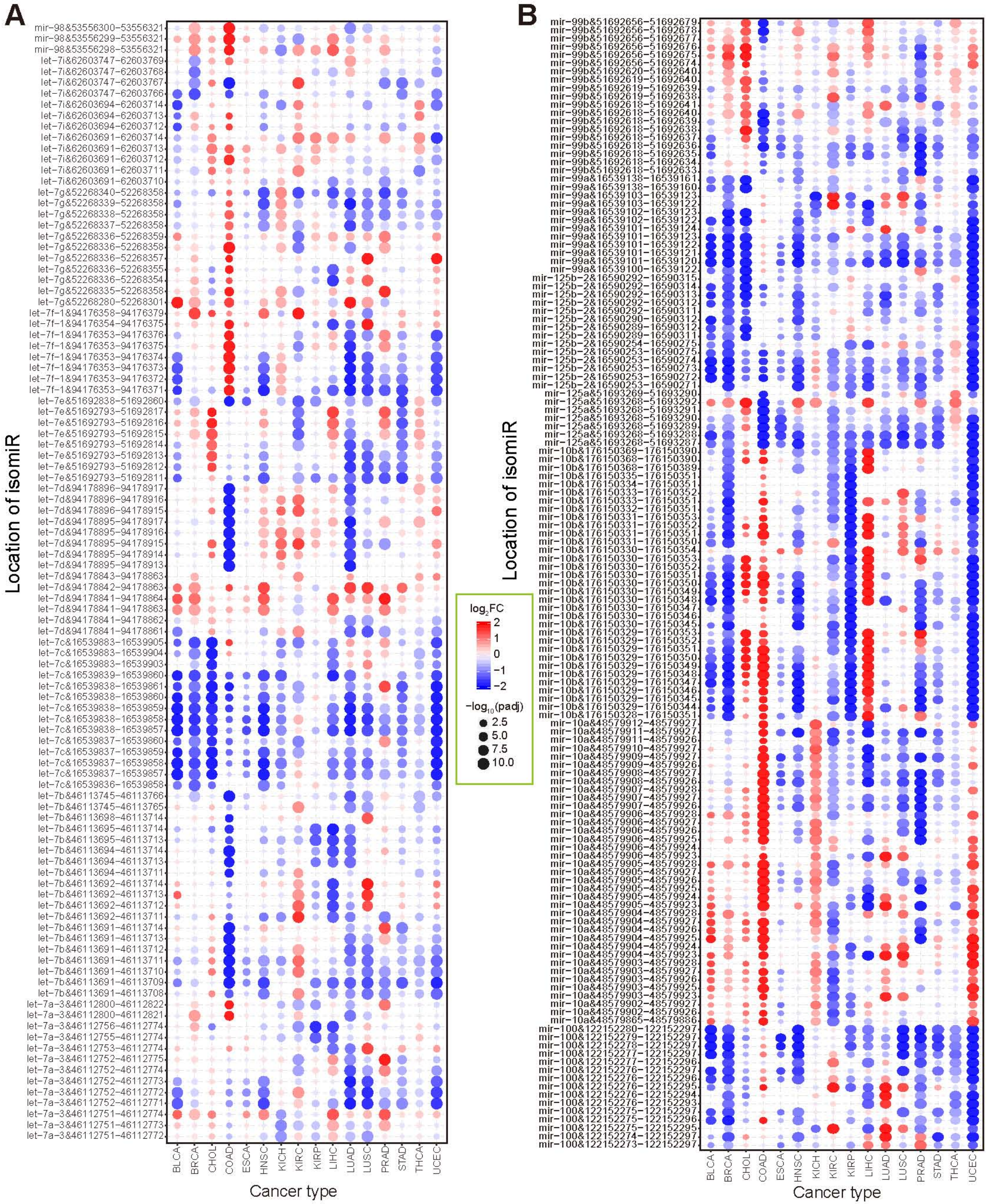

A
B
Location of isomiR
Cancer type
BLCA
BRCA
CHOL
COAD
ESCA
HNSC
KICH
KIRC
KIRP
LIHC
LUAD
LUSC
PRAD
STAD
THCA
UCEC
log2FC
2
1
0
−1
−2
−log10(padj)
2.5
5.0
7.5
10.0

Figure S1. Expression patterns of miRNAs from the let-7 and miR-10 gene families. (**A**) Distribution of isomiR types and seed numbers across different miRNA loci for the two gene families (isomiRs with RPM ≥ 50 were analyzed) across diverse cancer types. No significant differences in the number of isomiRs were detected between the tumor and normal samples using a Wilcoxon rank-sum test (p = 0.4273 for the let-7 family and p = 0.4260 for the miR-10 family). The isomiR type and seed number were estimated according to the maximum value across different cancer types. Examples of distributions of isomiR type and seed number at several miRNA loci (let-7a-5p, let-7b-5p, miR-10a-5p, and miR-10b-5p) across diverse cancer types are shown. (**B**) Distribution of isomiR types and seed numbers across different miRNA loci for the two gene families in BRCA. (**C**) Box plots showing relative expression (%) of homologous miRNAs in BRCA (*n* = 1207). p-values were estimated using the median values based on trend test. Expression analysis was performed using expression data for each miRNA gene (miRNA sequencing data from TCGA). (**D**) Examples of heat maps showing the distribution of expression of homologous miRNAs in BRCA (*n* = 1207) using $\log_2$RPM values for the two gene families. Tumor and normal samples are simultaneously presented.

Figure S2

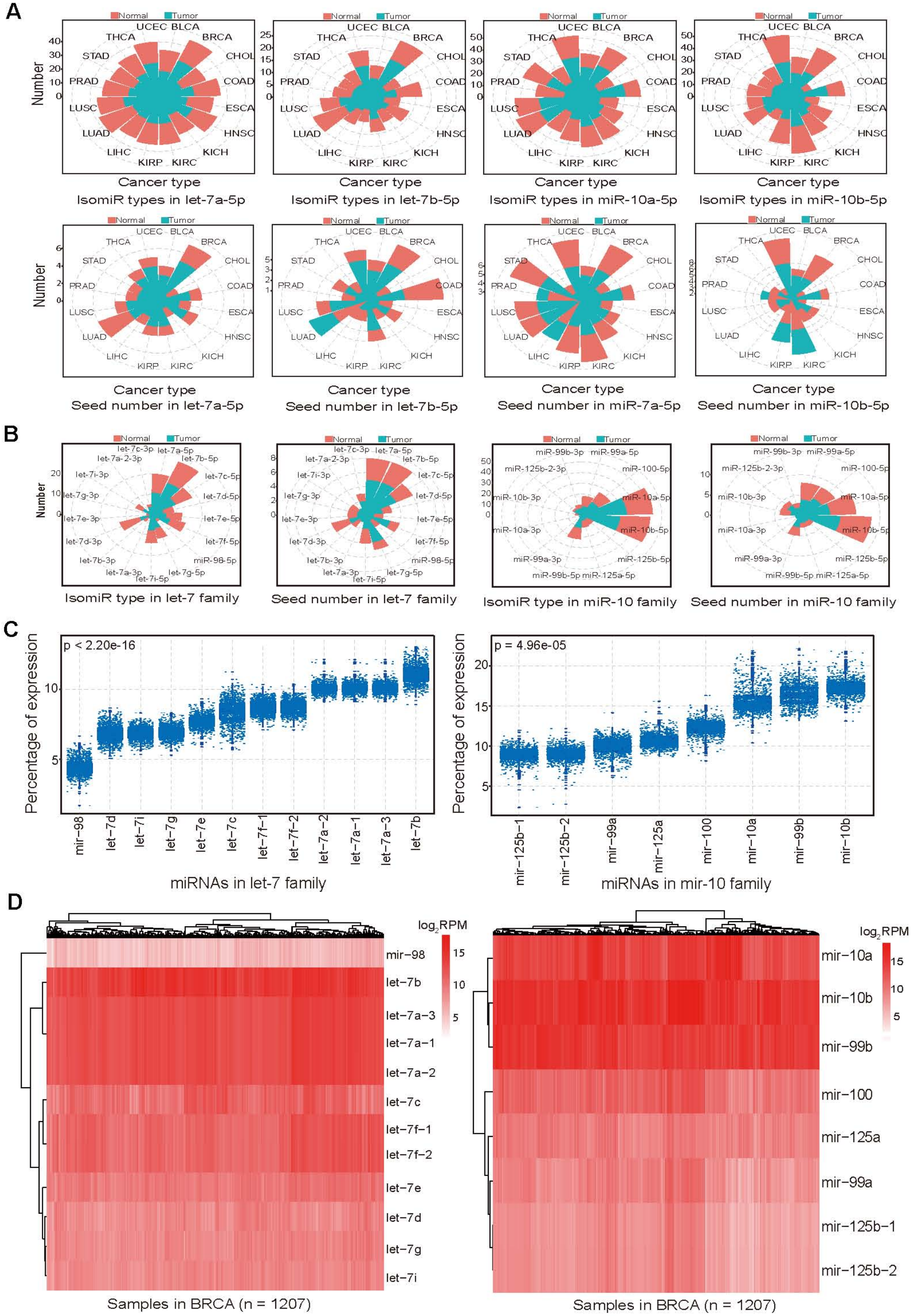
A
Normal
Tumor
Number
Cancer type
IsomiR types in let-7a-5p
IsomiR types in let-7b-5p
IsomiR types in miR-10a-5p
IsomiR types in miR-10b-5p
Seed number in let-7a-5p
Seed number in let-7b-5p
Seed number in miR-7a-5p
Seed number in miR-10b-5p
B
IsomiR type in let-7 family
Seed number in let-7 family
IsomiR type in miR-10 family
Seed number in miR-10 family
C
p < 2.20e-16
p = 4.96e-05
Percentage of expression
miRNAs in let-7 family
miRNAs in mir-10 family
D
log2RPM
Samples in BRCA (n = 1207)
Samples in BRCA (n = 1207)

Figure S2. Expression patterns of isomiRs from the let-7 and miR-10 gene families. (**A**) Dysregulated expression patterns of isomiRs from the let-7 gene family. Only isomiRs with a RPM ≥ 50 for least in one cancer type were included. (**B**) Dysregulated expression patterns of isomiRs from the mir-10 gene family.

Figure S3

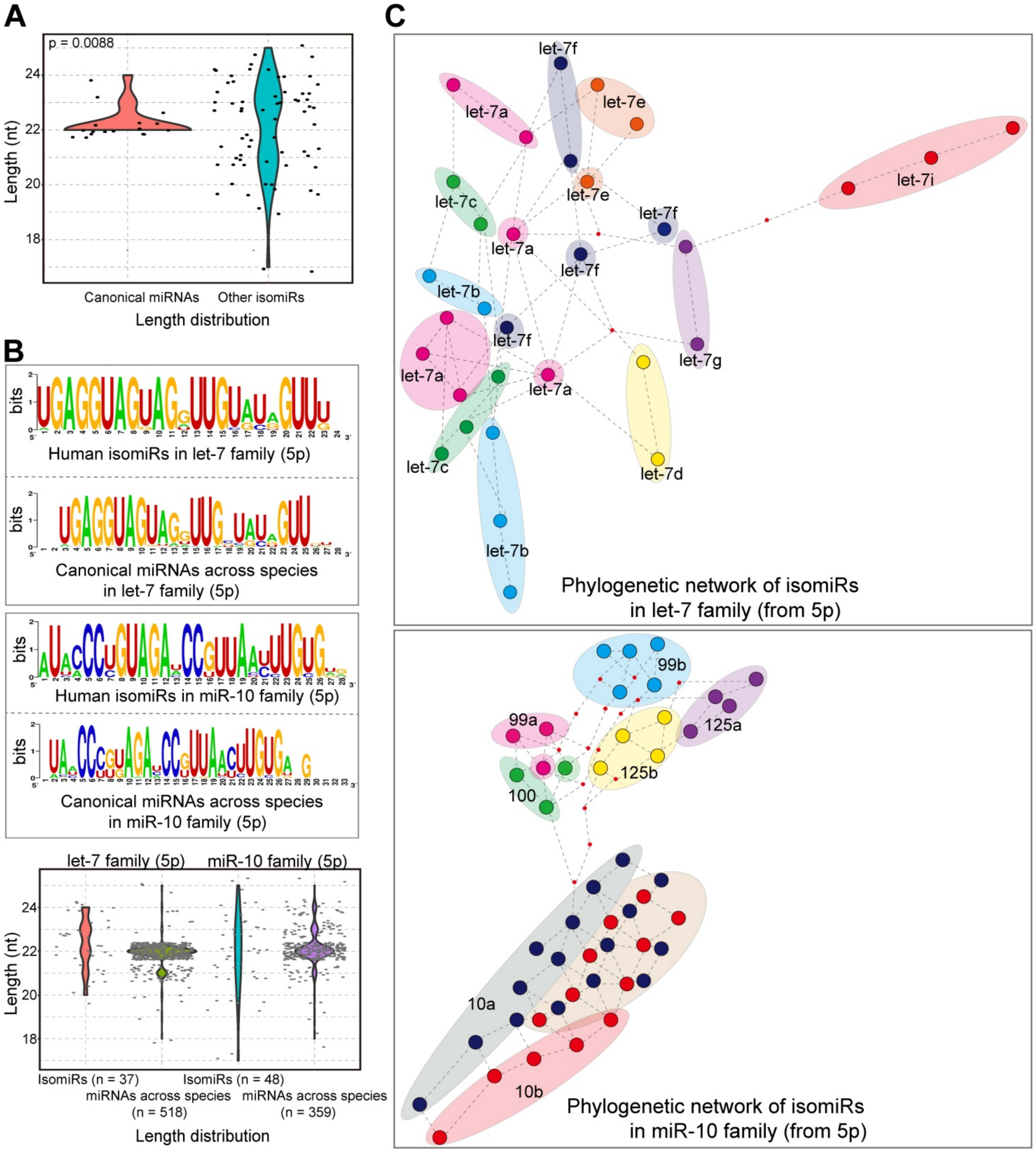

A
p = 0.0088
Length (nt)
Canonical miRNAs
Other isomiRs
Length distribution
B
Human isomiRs in let-7 family (5p)
Canonical miRNAs across species in let-7 family (5p)
Human isomiRs in miR-10 family (5p)
Canonical miRNAs across species in miR-10 family (5p)
let-7 family (5p)
miR-10 family (5p)
IsomiRs (n = 37)
miRNAs across species (n = 518)
IsomiRs (n = 48)
miRNAs across species (n = 359)
Length distribution
C
Phylogenetic network of isomiRs in let-7 family (from 5p)
Phylogenetic network of isomiRs in miR-10 family (from 5p)

Figure S3. Relationships among multiple isomiRs and canonical miRNA sequences. (**A**) Length distribution analysis shows variations in the lengths of the isomiRs compared with their canonical miRNAs (isomiRs were selected based on Figure 2C). Wilcoxon test results are shown. (**B**) Sequence features of isomiRs from 5p (obtained from Figure 2C) belonging to the two gene families, and sequence features of canonical miRNAs across different animal species. (**C**) Median-joining network for the isomiRs (based on Figure 2C) from the two gene families (from 5p).

Figure S4

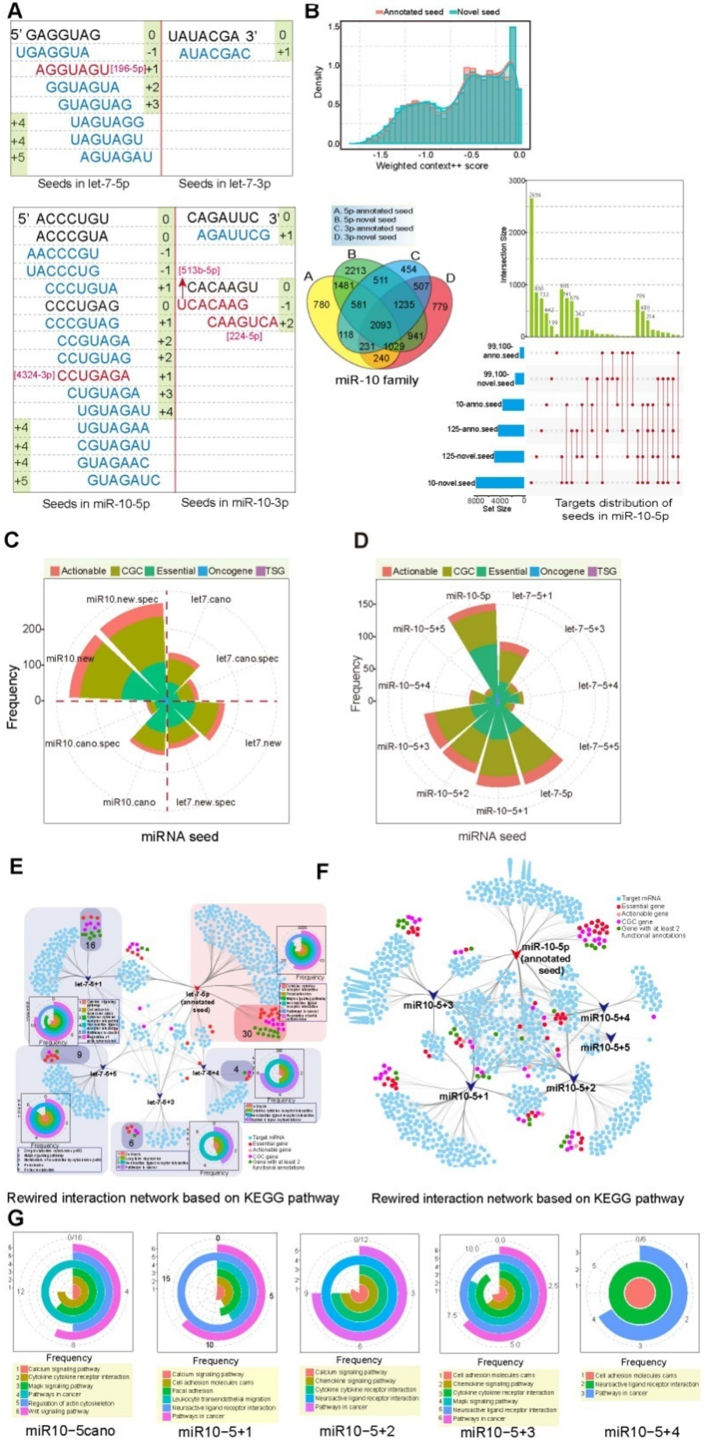

Figure S4. Seed variation across the multiple isomiRs and functional analysis of their predicted targets. (**A**) Novel seeds based on isomiR expression profiles for the two gene families. Seeds showing identity to seed sequences from other miRNAs are shown in red font. (**B**) Score distribution of predicted targets between novel seeds and annotated seeds, and distribution of target numbers for the mir-10 gene family. (**C**) Distribution of enriched cancer hallmarks among annotated and novel seeds. (**D**) Distribution of functional target genes among different seeds, mainly including actionable, CGC, core essential, oncogene, and TSG genes. (**E**) Rewired isomiR-mRNA interaction network based on target genes associated with KEGG pathways among seeds from the let-7-5p locus. Frequencies of associated genes in the KEGG pathways are also shown for specific targets of each seed. (**F**) Rewired isomiR-mRNA interaction network based on target genes associated with KEGG pathways among seeds from the miR-10-5p locus. (**G**) Top enriched KEGG pathways based on the number of associated genes among different seeds from Supplementary Figure S4E.

Figure S5

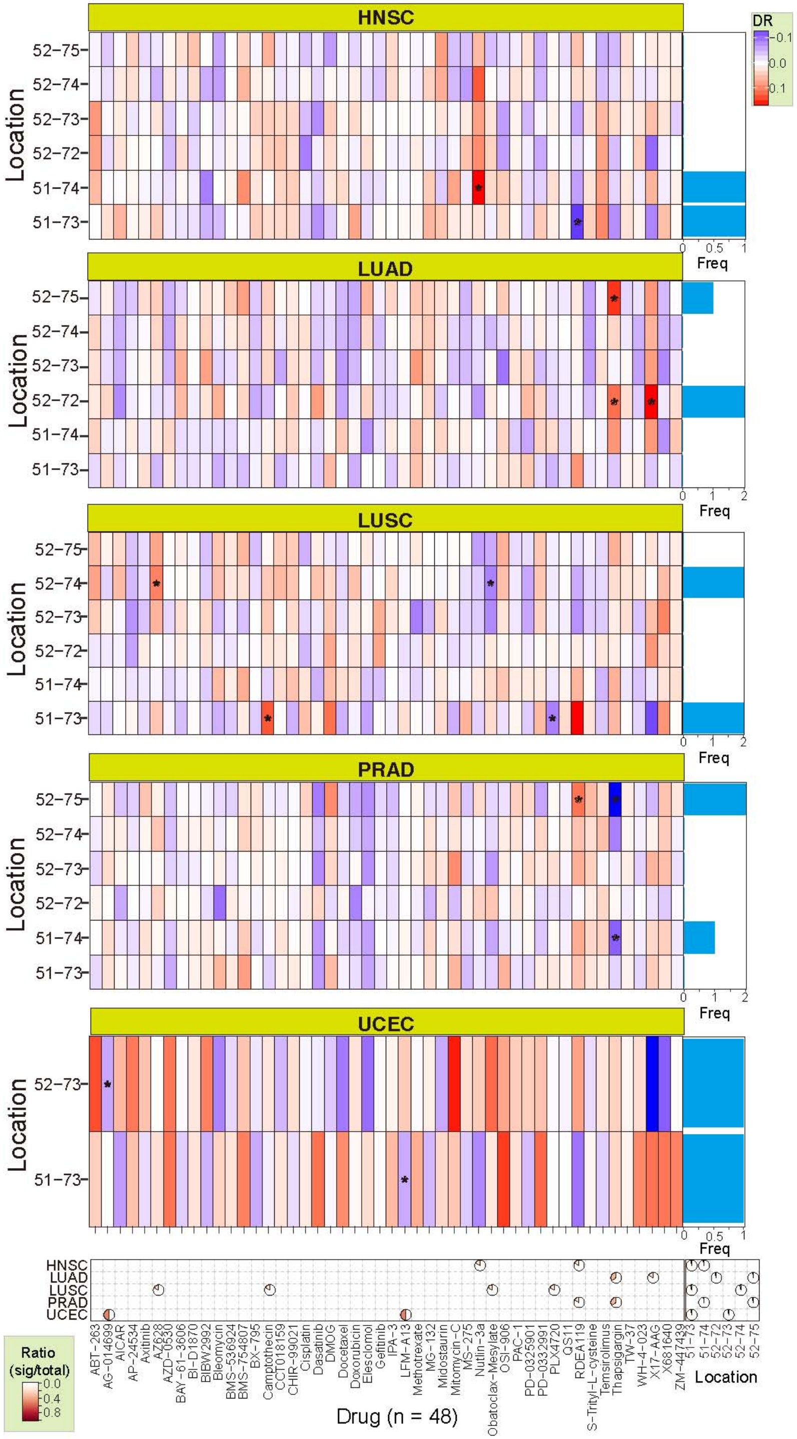

HNSC
LUAD
LUSC
PRAD
UCEC
Location
52−75
52−74
52−73
52−72
51−74
51−73
Freq
DR
−0.1
0.0
0.1
Ratio (sig/total)
0.0
0.4
0.8
ABT−263
AG−014699
AICAR
AP−24534
Axitinib
AZ628
AZD−0530
BAY−61−3606
BI−D1870
BIBW2992
Bleomycin
BMS−536924
BMS−754807
BX−795
Camptothecin
CCT018159
CHIR−99021
Cisplatin
Dasatinib
DMOG
Docetaxel
Doxorubicin
Elesclomol
Gefitinib
IPA−3
LFM−A13
Methotrexate
MG−132
Midostaurin
Mitomycin−C
MS−275
Nutlin−3a
Obatoclax−Mesylate
OSI−906
PAC−1
PD−0325901
PD−0332991
PLX4720
QS11
RDEA119
S−Trityl−L−cysteine
Temsirolimus
Thapsigargin
TW−37
WH−4−023
X17−AAG
X681640
ZM−447439
Drug (n = 48)

Figure S5. Drug responses for different isomiRs of the let-7a-5p locus. Drug responses associated with six isomiRs (two seeds) of the let-7a-5p locus based on data from Figure 5C. Asterisks (*) indicate drugs for which a significant difference was observed between the abnormal expression and normal expression groups ($DR > 0.1$ or $DR < -0.1$ and $p < 0.05$). Among the six isomiRs, only four were detected in CHOL. “DR” indicates a difference in the values of the shifted seed group and the annotated seed group. “Freq” indicates frequency. “Sig/total” indicates the ratio of the number of significant results and the total number of isomiRs (pie distribution, left) or the total number of drugs (pie distribution, right). The location annotation provides a detailed locus for each isomiR. For example, 51-73 indicates the location hg38:chr22:46112751-46112773:+.
